# Supplementary material for: Binding of regulatory proteins to nucleosomes is modulated by dynamic histone tails
Source: Nat Commun. 2021 Sep 6;12:5280. doi: 10.1038/s41467-021-25568-6 (PMC8421395; doi:10.1038/s41467-021-25568-6)
Supplement: Supplementary file 1 — Supplementary Information [file 41467_2021_25568_MOESM1_ESM.docx]

**Binding of regulatory proteins to nucleosomes is modulated by dynamic histone tails**

Yunhui Peng^1^, Shuxiang Li^2^, Alexey Onufriev^3,4,5^, David Landsman^1^ and Anna R. Panchenko^2*^

^1^National Center for Biotechnology Information, National Institutes of Health, Bethesda, MD, USA

^2^ Department of Pathology and Molecular Medicine, School of Medicine, Queen’s University, ON, Canada

^3^Physics Department, Virginia Tech, VA, USA

^4^Computer Science Department, Virginia Tech, VA, USA

^5^Center for Soft Matter and Biological Physics, Virginia Tech, VA, USA

*- corresponding author, anna.panchenko@queensu.ca

**Supplementary Note 1**

***Simulation protocols details***

In the beginning of this study, we performed short simulation runs using CHARMM potentials and TIP3P water models which we decided to report in the paper for a purpose of providing a more complete picture (see Supplementary Table 1). Previous work has shown that the TIP3P water model may lead to the overly compact conformations of intrinsically disordered regions, like histone tails (IDP), regardless of the underlying gas-phase force-field ^1^. While a specialized water model TIP4P-D was recently developed to address this defect in simulations of IDPs ^1^, a general-purpose water model, OPC, has been shown to perform very well on histone tails ^2^ and IDPs in general ^3^. Besides proteins, OPC water model has been extensively tested in simulations of RNA ^4-6^ and DNA ^7,8^. Most recently, OPC water model has been applied to simulate the interactions between the linker histone globular domains and DNA in the context of the chromatosome ^9^.

Using the OPC water model, we observed a much slower histone tail condensation on the nucleosomal and linker DNA on the timescale of ~100 ns compared to 10-50 ns using the TIP3P water model. One possible reason for these differences could arise from self-diffusion coefficient values used for the TIP3P water model being about 2.5 times larger than the experimental values, which could artificially accelerate the motions in the simulations. Simulations using the OPC water model show many rapid interconversions between tail-DNA bound and unbound states, pointing to a more dynamic histone tail behavior compared to simulations with the TIP3P water model where histone tails remain in the bound state with DNA most of the time ^10,11^ (Figure 1). The reason could be that the TIP3P water model can over-stabilize the compact states compared to extended conformations ^3^ and therefore histone tails are rarely observed in unbound states after their initial condensation ^11,12^. Therefore, throughout the paper for all our analyses we only used simulations with the OPC water model.

Simulations using CHARMM forcefields and TIP3P water models were prepared with VMD ^13^ and performed using NAMD 2.12 package ^14^. The systems were initially subjected to 1000 steps of energy minimization with all protein and DNA atoms fixed and then to another 10,000 steps of minimization without constraints. Next, we performed four rounds of 200 ps equilibrations with elastic constraints on C-α atoms of protein and P atoms of DNA backbone, which were gradually relaxed as follows: 90 -> 45-> 9-> 0 kcal/mol/A^2^.

**Supplementary Note 2**

***Theoretical models to predict the effective binding free energy of partners to nucleosomes***

To elucidate how histone tails may modulate the binding affinity of a binding partner (“Pro”) to nucleosome, we offer theoretical models to predict the effective binding free energy of partners to nucleosomal DNA with the tails involved according to the following two scenarios.

First, we predict the effective binding constant of a partner bound to nucleosome using thermodynamic cycle (Supplementary Fig. 15). We use the following experimentally measured values of dissociation constants from a recent study^15^ for binding of histone demethylase LSD1-CoREST to the nucleosome (measured at zero salt concentration so could be considered as an upper bound for the binding energy estimates): binding free energy between H3 tail and LSD1-CoREST, ΔG_0_(Tail-Pro) = -9.9 kcal/mol; binding energy between H3 tail and DNA (nucleosome), ΔG_0_(Tail-DNA) = -11.14 kcal/mol; and binding energy between LSD1-CoREST and free DNA, ΔG_0_(Pro-DNA) = -9.75 kcal/mol. Based on these values we can estimate the effective binding free energy of a binding partner to nucleosome as: ΔG_0_(Pro-Tail-DNA) = ΔG_0_(Pro-DNA) + ΔG_0_(Tail-Pro) - ΔG_0_(Tail-DNA) = -9.75 – 9.9 + 11.14 = -8.51 kcal/mol, where ΔG_0_(Pro-Tail-DNA), ΔG_0_(Tail-DNA), ΔG_0_(Pro-DNA), and ΔG_0_(Tail-Pro) represent standard binding free energies of a partner to the tail-DNA complex (nucleosome), of histone tails to the DNA (nucleosome), of a partner to the free DNA, and of histone tails to binding partners respectively. As one can see from the thermodynamic cycle and this equation, tail-partner interactions may contribute positively to the overall binding of a partner to nucleosome while tail-DNA interactions contribute negatively and interfere with the interactions between a partner and nucleosome.

If we do not consider tail-partner interactions, in case they are highly unfavorable for binding each other, then we can calculate the selectivity constant K_pt_ to describe binding of a partner to a tail-DNA complex (in a nucleosome) in 1:1 stoichiometry.

${Pro+DNA \leftrightarrow Pro-DNA; K}_{\mathrm{pa}}= \frac{[Pro-DNA]}{\left[ \mathrm{Pro} \right][DNA]}$ (1)

${Tail+DNA\leftrightarrow Tail-DNA; K}_{\mathrm{ta}}= \frac{[Tail-DNA]}{\left[ \mathrm{Tail} \right][DNA]}$ (2)

$Tail-DNA+Pro \leftrightarrow Pro-DNA+Tail;K_{\mathrm{pt}}= \frac{[Pro-DNA][Tail]}{[Tail-DNA]\left[ \mathrm{Pro} \right]}=\frac{[Pro-DNA]}{\left[ \mathrm{Pro} \right][DNA]}*\frac{\left[ \mathrm{Tail} \right][DNA]}{[Tail-DNA]} = \frac{K_{\mathrm{pa}}}{K_{\mathrm{ta}}}$ (3)

Given the favorable association of histone tails with DNA (K_ta_ > 1), $K_{\mathrm{pt}}$ , equilibrium constant for the reaction of the tail displacement by the nucleosome binding partner would be smaller than the association constant of a partner to the free DNA. This implies that in cases where histone tails and partner occupy overlapping regions on nucleosomal DNA (Figure 3d) – it can considerably decrease the binding of partners to nucleosomes. For example, in this work, our estimates of the association constant $K_{\mathrm{ta}}$ for H3 and H4 tails with DNA are about 100 to 1,000 $M^{-1}$ (Supplementary Table 4). $K_{\mathrm{pa}}$ for binding of a partner to the free DNA can vary between 10^4^ to 10^11^ $M^{-1}$ (dbAMEPNI database^16^, Supplementary Fig. 23).

The local concentrations of histone tails and nucleosome binding partners are several orders of magnitude different in a cell^17^. For instance, the concentrations of transcription factors (TFs) inside the human cell are usually in the nM to µM range ^18,19^. In contrast, a human cell contains about 10^7^ copies of histones each. Moreover, this is a lower bound estimate because the local concentration of histones and histone tails in chromatin is even higher since they are tethered on nucleosomes. The standard binding energies values of nucleosome binding partners should be corrected by their actual relatively low concentrations in a cell, resulting in even lower effective binding affinities to nucleosomal/linker DNA.

**Supplementary Table 1.** Summary of simulations for modeling of histone tail conformational ensembles in the context of the full nucleosome. Throughout the paper for all our analyses we used simulations with the AMBER force field and OPC water model.

| Force field | Initial Tail conformations | DNA sequence | Simulation PACKage | Simulation time |
| --- | --- | --- | --- | --- |
| AMBER FF14SB and OL15 + OPC water model | **Model A:**  Template from PDB 1aoi and 1kx5 | 187 bp DNA sequence (including the linker DNA) from the *KRAS* gene, +1 nucleosome | AMBER | 1 run *4500ns  4 runs * 800ns |
|  | **Model B:**  Template from PDB 1eqz and 1kx5 |  | AMBER | 1 run *5000ns  4 runs * 800ns |
|  | **Model C:**  Extended tail configurations |  | AMBER | 1 run *4500ns  4 runs * 800ns |
|  | **Model D:**  Symmetrically extended tail configurations |  | AMBER | 1 run *4000ns  4 runs * 800ns |
|  | **Model D:**  Symmetrically extended tail configurations |  | GROMACS | 1 run *5000ns |
|  | **Model D:**  Symmetrically extended tail configurations |  | GROMACS | 1 run *5000ns |
| CHARMM36m+ TIP3P water model | **Model A:**  Template from PDB 1aoi and 1kx5  **Model B:**  Template from PDB 1eqz and 1kx5  **Model C:**  Extended tail configurations  **Model D:**  Symmetrically extended tail configurations |  | NAMD | Total 1200ns |

**Supplementary Table 2.** Summary of simulations for modeling of mutated or post-translationally modified histone tails. The simulated post-translational modifications or mutations include Lys acetylation, Lys tri-methylation, Ser/Thr phosphorylation, and Arg -> Ala mutation. Several modifications or mutations of the same type are introduced at the same time in the simulation sets 1-4, while modifications or mutations are introduced at one residue at a time in the simulation set 5.

|  | Modified Residues | Initial Tail conformations | Forcefield | SIMULATION PACKAGE | Simulation time |
| --- | --- | --- | --- | --- | --- |
| Simulation Set 1  Lys acetylation | H2A-N: K5, K9  H2A-C: K126  H2B: K2, K9, K12, K17, K20  H3: K4, K9, K14, K23, K27, K36  H4: K5, K8, K12 | **Model D:**  Symmetrically extended tail configurations | AMBER FF14SB and OL15 + OPC water model | AMBER | 1 run *1600ns  4 runs * 800ns |
| Simulation Set 2  Lys Methylation (Kme3) | H2A-N: K9  H2A-C: K124  H2B: K2, K9, K12, K17, K20  H3: K4, K9, K14, K23, K27, K36  H4: K5, K12 |  |  | AMBER | 1 run *1600ns  4 runs * 800ns |
| Simulation Set 3  SER/THR Phosphorylation | H2A-N: S1  H2B: S3, S11  H3: T3, S10, S28  H4: S1 |  |  | AMBER | 1 run *1600ns  4 runs * 800ns |
| Simulation Set 4  Arg -> Ala Mutation | H2A-N: R3, R11  H3: R2, R8, R17, R26  H4: R3 |  |  | AMBER | 1 run *1600ns  4 runs * 800ns |
| Simulation Set 5  Single modification per tail | H2A-N: S1(ph)  H2A-C: K126(ac)  H2B: K2(ac)  H3: R2A  H4: K5(ac) |  |  | AMBER | 1 run *1600ns  4 runs * 800ns |

**Supplementary Table 3.** Definitions of histone alpha-helical core and tail regions, following our previous study ^12^. For estimating the full histone tail residence time on DNA, we exclude those tail residues that are located within 8 Å of DNA molecules in extended tail configurations.

| Histone regions | Definitions |
| --- | --- |
| Alpha helices in histone core | H2A: residue 27 to 37, 45 to 73, 80 to 89  H2B: residue 34 to 45, 53 to, 88 to 98  H3: residue 64 to 78, 86 to 114, 121 to 131  H4: residue 31 to 41, 49 to 76, 83 to 93 |
| H2A N- and C-terminal tail | Residue 1 to 13 and 119 to 128 |
| H2B tail | Residue 1 to 23 |
| H3 tail | Residue 1 to 36 |
| H4 tail | Residue 1 to 20 |
| Tail residues for full histone tail residence time calculation and MM/PBSA calculation | H2A N- and C-terminal tail: residue 1 to 8 and 120 to 128 |
|  | H2B tail: residue 1 to 18 |
|  | H3 tail: residue 1 to 33 |
|  | H4 tail: residue 1 to 15 |

**Supplementary Table 4.** Estimating the histone tail dissociation constant and standard binding free energy with DNA from histone tail conformational ensemble statistics. Histone tail unbound state is defined if the percentage of tail residues maintaining contacts with the DNA molecule is no more than the “cut-off” value. The dissociation constant ($K_{d}$) can be estimated by $K_{d}=\frac{number of frames in unbound state}{number of frames in bound state}$ for each histone type. Then, the histone tail’s binding free energy with DNA (ΔG_0_ in kcal/mol) is derived from $K_{d}$ using the following equation: $G_{0}=RT\ln K_{d}$. For each simulation run, we count the number of observed unbinding events of each tail copy and only include those runs that have at least five unbinding events for the analysis. “NA” indicates that more than five unbinding events were not observed in simulation runs using the chosen cut-off. Binding free energy values (ΔG_0_) derived from the tail conformational ensemble statistics generally decrease as cut-off values increase. High correlation coefficients are retained between ΔG_0_ estimated from MM/GBSA calculations and histone tail conformational ensemble statistics for different parameter values (Supplementary Fig. 4).

|  | **Number of frames in unbound state** | **Number of frames in bound state** | **Cut-off** | $\boldsymbol{K}_{\boldsymbol{d}}$**(M)** | **ΔG_0_ (kcal/mol)** |
| --- | --- | --- | --- | --- | --- |
| **H2A N-Tail** | 6642 | 175342 | 0 | 0.038 | -2.02 |
| **H2A C-Tail** | 27345 | 240631 | 0 | 0.11 | -1.34 |
| **H2B Tail** | 671 | 130817 | 0 | 0.0051 | -3.25 |
| **H3 Tail** | NA | NA | 0 | NA | NA |
| **H4 Tail** | 195 | 47801 | 0 | 0.0041 | -3.39 |
|  |  |  |  |  |  |
| **H2A N-Tail** | 6642 | 175342 | 0.1 | 0.038 | -2.02 |
| **H2A C-Tail** | 27345 | 240631 | 0.1 | 0.11 | -1.34 |
| **H2B Tail** | 3024 | 171460 | 0.1 | 0.018 | -2.49 |
| **H3 Tail** | 29 | 23969 | 0.1 | 0.0012 | -4.14 |
| **H4 Tail** | 2697 | 176787 | 0.1 | 0.015 | -2.58 |

**Supplementary Table 5.** Estimating the histone tail dissociation constant using MM/GBSA approach. Dissociation constants ($K_{d}$) of histone tails are estimated using the following equation: $K_{d}= exp(\frac{G_{0}}{RT})$. An average binding free energy per tail residue is calculated as the total tail binding free energy divided by the number of tail residues. Note that MM/GBSA approach tends to overestimate the absolute energy values but can be used for ranking of tails with respect to their binding energy.

|  | Tail-DNA binding free energy (kcal/mol) | Average binding free energy per tail residue (kcal/mol) | Dissociation constant(M) |
| --- | --- | --- | --- |
| H2A N-Tail | -13.80 | -1.73 | 1.86 E-10 |
| H2A C-Tail | -7.21 | -0.80 | 8.29E-06 |
| H2B Tail | -21.78 | -1.21 | 4.44E-16 |
| H3 Tail | -52.50 | -1.59 | 9.62E-38 |
| H4 Tail | -18.40 | -1.23 | 1.06E-13 |

**Supplementary Table 6.** Analysis of variance (ANOVA) and Tukey HSD test for histone tail residence time among different tail types. The null hypothesis is that there is no difference in the means of residence time between different histone tail groups. Two-tailed ANOVA F-test and one-tailed Tukey HSD test were performed.

| ANOVA | P-value = 7.93E-33 | | F-value = 42.73845 | |
| --- | --- | --- | --- | --- |
| **Tukey HSD test (multiple pairwise comparison)** | | | | |
| **group1** | **group2** | **﻿meandiff** | **adjusted p-values** | **﻿NULL hypothesis** |
| H2A_C | H2A_N | 172.1319 | 0.0393 | rejected |
| H2A_C | H2B | 180.6926 | 0.0266 | rejected |
| H2A_C | H3 | 1607.7515 | 0.001 | rejected |
| H2A_C | H4 | 207.2717 | 0.0089 | rejected |
| H2A_N | H2B | 8.5608 | 0.9 | not rejected |
| H2A_N | H3 | 1435.6196 | 0.001 | rejected |
| H2A_N | H4 | 35.1399 | 0.9 | not rejected |
| H2B | H3 | 1427.0588 | 0.001 | rejected |
| H2B | H4 | 26.5791 | 0.9 | not rejected |
| H3 | H4 | -1400.4798 | 0.001 | rejected |

**Supplementary Table 7.** The Pearson correlation coefficients between the mean number of tail-DNA contacts at each DNA base pair calculated for different nucleosome models. Two-tailed t-test is performed.

|  | ModelA | ModelB | ModelC | ModelD |
| --- | --- | --- | --- | --- |
| ModelA | 1 | 0.71 | 0.66 | 0.58 |
| ModelB | 0.71 | 1 | 0.71 | 0.6 |
| ModelC | 0.66 | 0.71 | 1 | 0.8 |
| ModelD | 0.58 | 0.6 | 0.8 | 1 |

**Supplementary Table 8.** PDB IDs of nucleosome complex structures where nucleosome-binding proteins interact with both DNA and histone tails.

| H3 tail and DNA | 6NZO, 6PX3, 6R1U, 6S01, 6UGM, 6UH5, 6VEN, 6VYP, 6WKR, 7AT8, 7CRO, 7CRP, 7CRQ, 7CRR |
| --- | --- |
| H4 tail and DNA | 3TU4, 5O9G, 5X0Y, 5Z3L, 5Z3O, 5Z3U, 5Z3V, 6FTX, 6G0L, 6IRO, 6IY2, 6IY3, 6JYL, 6K1P, 6KIV, 6KW3, 6KW4, 6MUO, 6MUP, 6NE3, 6PWF, 6RYR, 6RYU, 6KW5, 6VZ4, 6W5N, 7K6P, 7K6Q, 7BXT |
| H2A C-tail and DNA | 7CRO, 7CRP, 7CRQ, 7CRR |

**Supplementary Table 9.** Experimentally measured dissociation constant$(K_{d}$) and standard binding free energy (ΔG_0_) of proteins bound to mononucleosomes. The experimentally determined $K_{d}$ (in nM) is taken from a recent quantitative mass spectrometry measurement ^20^, and ΔG_0_ (in kcal/mol) is derived using the equation: $G_{0}=RT\ln K_{d}$.

| Gene name | $\mathbf{K}_{\mathbf{d}}\boldsymbol{(nM)}$ | ΔG(kcal/mol) | Gene name | $\mathbf{K}_{\mathbf{d}}\boldsymbol{(nM)}$   \|  \| \| --- \| | ΔG(kcal/mol) |
| --- | --- | --- | --- | --- | --- | --- |
| CHD1L | 56.40 | -9.90 | **APTX** | 198.12 | -9.15 |
| KDM2A | 58.42 | -9.88 | **LIG3** | 209.01 | -9.12 |
| XRCC5 | 70.67 | -9.76 | **XRCC1** | 210.12 | -9.12 |
| XRCC6 | 76.67 | -9.72 | **PNKP** | 218.05 | -9.10 |
| RFC1 | 77.81 | -9.71 | **SCML2** | 221.74 | -9.09 |
| PARP2 | 80.65 | -9.69 | **RPA3** | 233.51 | -9.06 |
| RFC2 | 82.35 | -9.67 | **KMT2A** | 236.89 | -9.05 |
| RFC3 | 82.56 | -9.67 | **RPA1** | 247.50 | -9.02 |
| RFC5 | 84.56 | -9.66 | **PARP1** | 248.37 | -9.02 |
| RFC4 | 86.81 | -9.64 | **RPA2** | 264.89 | -8.98 |
| BPTF | 92.40 | -9.60 | **AHCTF1** | 270.40 | -8.97 |
| SMARCA5 | 109.83 | -9.50 | **DNTTIP1** | 283.70 | -8.94 |
| TREX1 | 123.92 | -9.43 | **MDC1** | 288.73 | -8.93 |
| SMARCA4 | 126.00 | -9.42 | **DDB2** | 298.85 | -8.91 |
| SMARCA2 | 126.35 | -9.42 | **TOP2B** | 307.36 | -8.89 |
| ACTL6A | 136.57 | -9.37 | **DDB1** | 308.08 | -8.89 |
| RAD23B | 137.74 | -9.37 | **TOP2A** | 316.98 | -8.87 |
| USF2 | 138.99 | -9.36 | **CCDC86** | 321.35 | -8.87 |
| CETN2 | 147.48 | -9.33 | **RIF1** | 330.68 | -8.85 |
| BAZ1B | 149.69 | -9.32 | **TFAM** | 346.16 | -8.82 |
| PMPCA | 153.48 | -9.30 | **ELMSAN1** | 349.36 | -8.82 |
| HLTF | 169.13 | -9.25 | **HMG20B** | 357.73 | -8.80 |
| ATF7 | 184.58 | -9.19 | **BANF1** | 371.95 | -8.78 |

**Supplementary Table 10.** DNA sequence of the *KRAS* gene +1 nucleosome.

| Name | Sequences |
| --- | --- |
| *KRAS* gene +1 nucleosome | 5' to 3' (chain J): CCCGCCCGGCGCCGGCAAAGAGGGTCGGGACCCGGGCAGGGGCCCAGGAGGGGTGGTCCGCTCCGTACCTCTCTCCCGCACCTGGGAGCCGCTGAGCCTCTGGCCCCGCCGCCGCCTTCAGTGCCTGCGCCGCGCTCGCTCCCAGTCCGAAATGGCGGGGGCCGGGAGTACTGGCCGAGCCGCCGCC  5' to 3' (chain I):  GGCGGCGGCTCGGCCAGTACTCCCGGCCCCCGCCATTTCGGACTGGGAGCGAGCGCGGCGCAGGCACTGAAGGCGGCGGCGGGGCCAGAGGCTCAGCGGCTCCCAGGTGCGGGAGAGAGGTACGGAGCGGACCACCCCTCCTGGGCCCCTGCCCGGGTCCCGACCCTCTTTGCCGGCGCCGGGCGGG |

**Supplementary Table 11.** PDB IDs of nucleosome complex structures.

| PDB ID | 1ZLA, 3MVD, 3TU4, 4JJN, 4KUD, 4LD9, 4R8P, 4X23, 4ZUX, 5E5A, 5GTC, 5HQ2, 5KGF, 5MLU, 5O9G, 5X0X, 5X0Y, 5Z3L, 5Z3O, 5Z3T, 5Z3U, 5Z3V, 6A5O, 6A5P, 6BUZ, 6C0W, 6DZT, 6E0C, 6E0P, 6FML, 6FTX, 6G0L, 6GEJ, 6GEN, 6HTS, 6I84, 6IRO, 6IY2, 6IY3, 6J4W, 6J99, 6JM9, 6JMA, 6JYL, 6K1P, 6KIU, 6KIV, 6KIW, 6KIX, 6KIZ, 6KW3, 6KW4, 6KW5, 6MUO, 6MUP, 6NE3, 6NJ9, 6NN6, 6NOG, 6NQA, 6NZO, 6O96, 6OM3, 6PA7, 6PWF, 6PWV, 6PWW, 6PWX, 6PX3, 6R1U, 6R25, 6R8Y, 6R8Z, 6R90, 6R91, 6R92, 6RYR, 6RYU, 6S01, 6SE6, 6SEE, 6SEF, 6T90, 6T9L, 6UGM, 6UH5, 6USJ, 6UXW, 6VEN, 6VYP, 6VZ4, 6W5I, 6W5M, 6W5N, 6WKR, 6X0N, 6X59, 6X5A, 6XJD, 6Y5D, 6Y5E, 6YOV, 6Z6P, 6ZHX, 6ZHY, 7A08, 7AT8, 7BWD, 7BXT, 7BY0, 7C0M, 7CCQ, 7CCR, 7CRO, 7CRP, 7CRQ, 7CRR, 7D1Z, 7D20, 7JO9, 7JOA, 7JZV, 7K5X, 7K5Y, 7K60, 7K61, 7K63, 7K6P, 7K6Q, 7K78, 7K7G |
| --- | --- |

**Supplementary Figure 1.** Four nucleosome models with different tail configurations. Nucleosome is solvated in the water box with Na+ and Cl- ions.


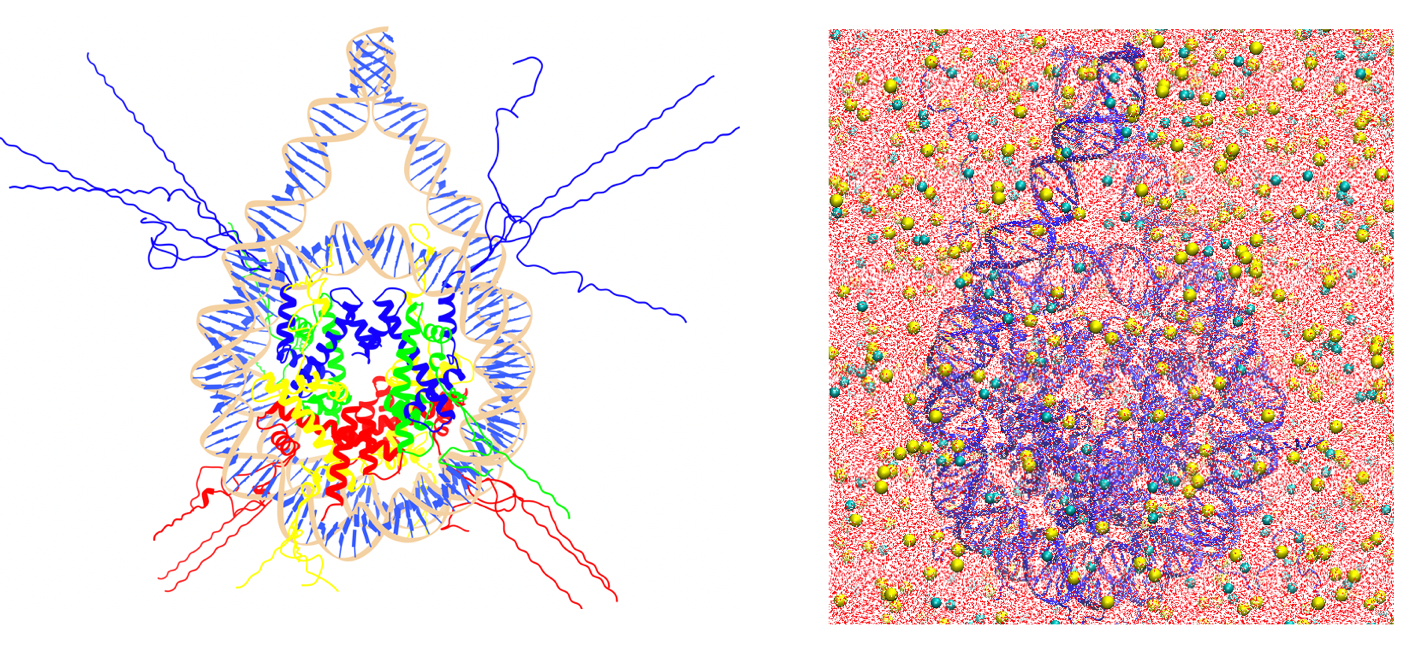


**Supplementary Figure 2.** Dependence of histone tail residence time ($\tau_{f}$) on the cut-off values for full tail unbound state definition. An unbound state is defined if the percentage of tail residues maintaining contacts with DNA is no more than the chosen cut-off. The results are combined from simulations of Models A, B, C and D, which have different initial tail configurations (described in Supplementary Table 1). We observe the same trend for cut-off = 0 and 0.1, where H3 tail has the longest residence time and the least number of unbinding events compared to other tails. Box-plot elements are defined as: center line, median; box limits, upper and lower quartiles; whiskers are drawn at values equal to 1.5× interquartile range; for cut-off = 0, n(H2A_N) = 174, n(H2A_C) = 359, n(H2B) = 91, n(H3) = 12, n(H4) = 52. For cut-off =0.1, n(H2A_N) = 174, n(H2A_C) = 359, n(H2B) = 173, n(H3) = 31, n(H4) = 160.


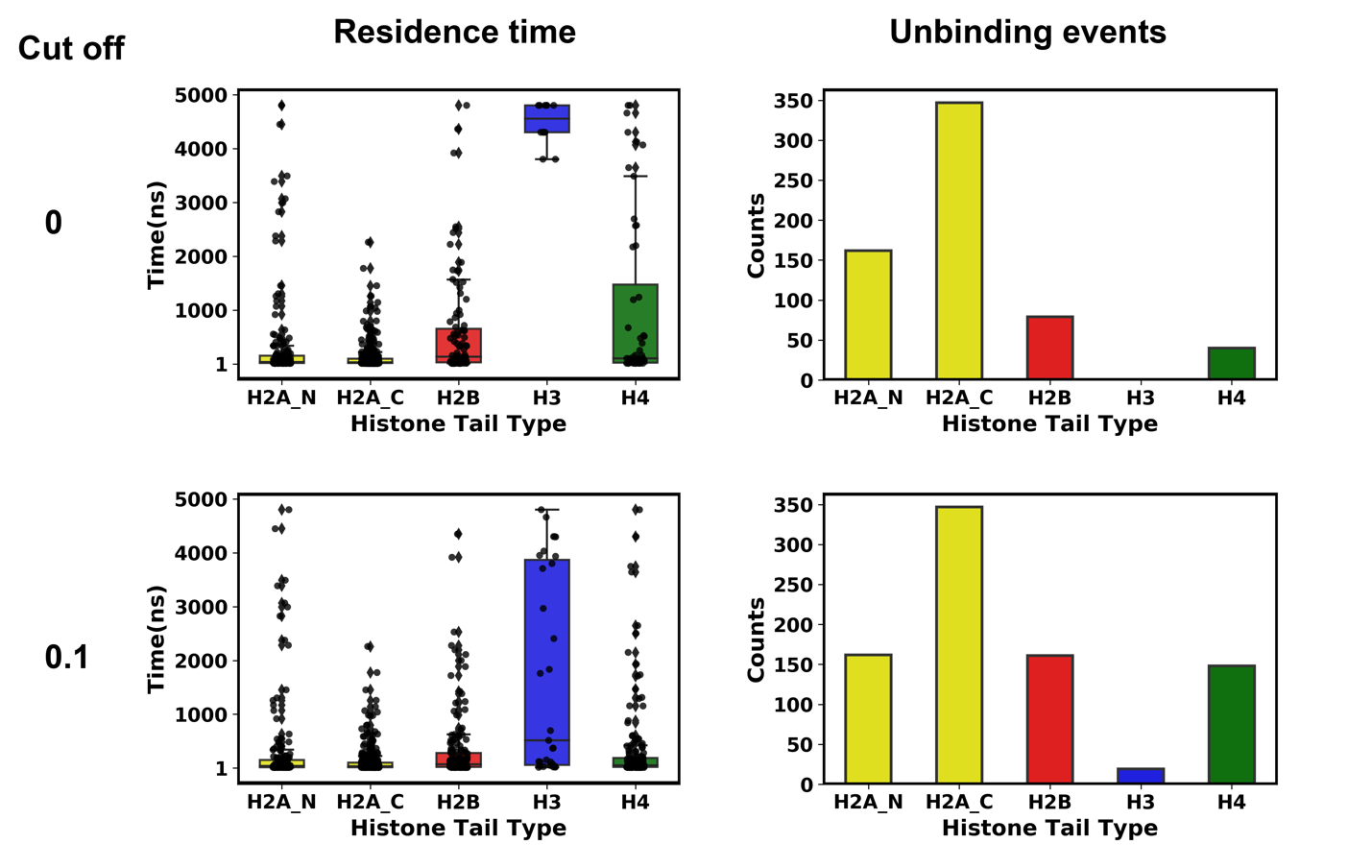


**Supplementary Figure 3.** Individual residue residence time ($\tau_{r}$) and binding free energy estimated by MM/GBSA approach. Residence time and binding energies are averaged and the error bars represent standard errors of the mean calculated from 22 independent simulation runs, each copy of tail is treated separately, so 44 data points in total. We ignore $\tau_{r}$ of H2A K13 calculated from one simulation run of Model B, where H2AK13 does not unbind from DNA during the simulation. Source data are provided as a Source Data file.

**
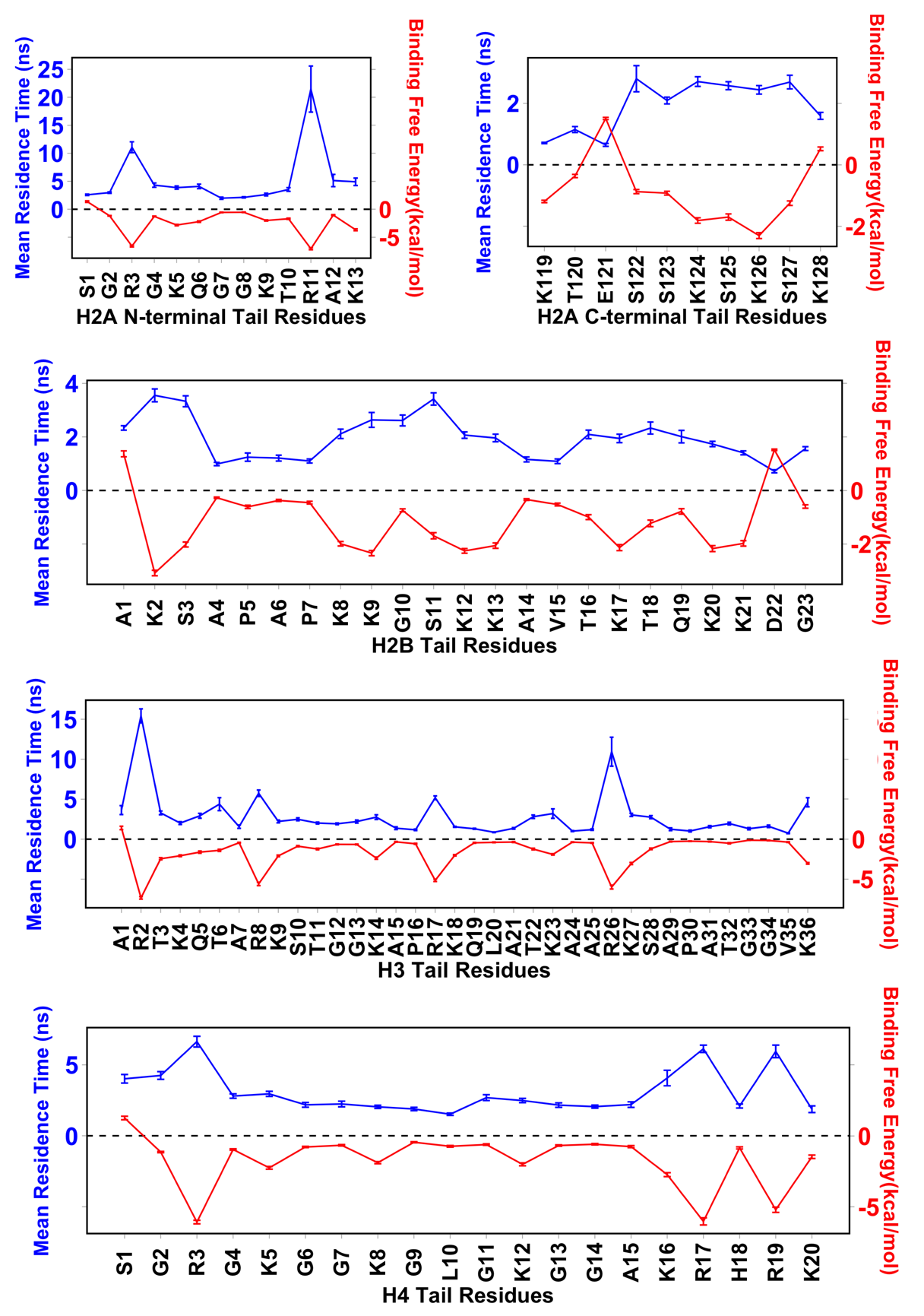
**

**Supplementary Figure 4.** Linear relationship between the histone tail-DNA binding free energy (ΔG_0_) derived from the tail conformational ensemble statistics (Supplementary Table 4) and MM/GBSA calculations (Supplementary Table 5). The Pearson correlation coefficients are calculated for two cut-offs in defining tail unbound states and thresholds on the number of observed unbinding events in simulation runs (described in Supplementary Table 4). ΔG_0_ values are in kcal/mol. We checked other different values of cut-off parameters and the resulting R^2^ > 0.9 for all cutoffs. Two-tailed t-test is performed.


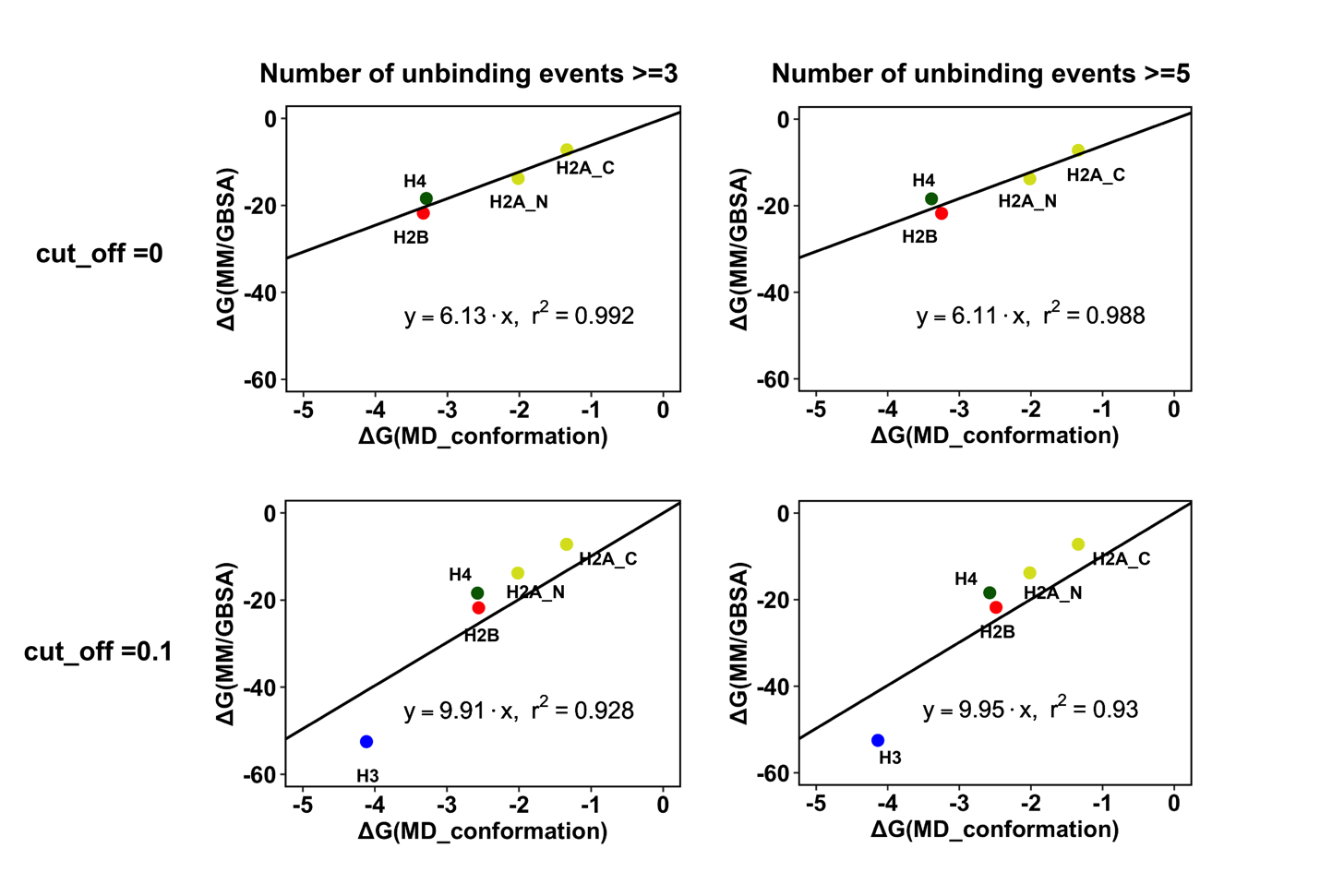


**Supplementary Figure 5.** Secondary structure propensity per tail residue plotted versus the percentage of frames where it was observed. Different types of secondary structures include Coil(C), Pi-Helix(I), 3-10 Helix(G), Alpha Helix(H), Isolated Bridge(B), Extended configuration(E), and Turn(T). Source data are provided as a Source Data file.

**
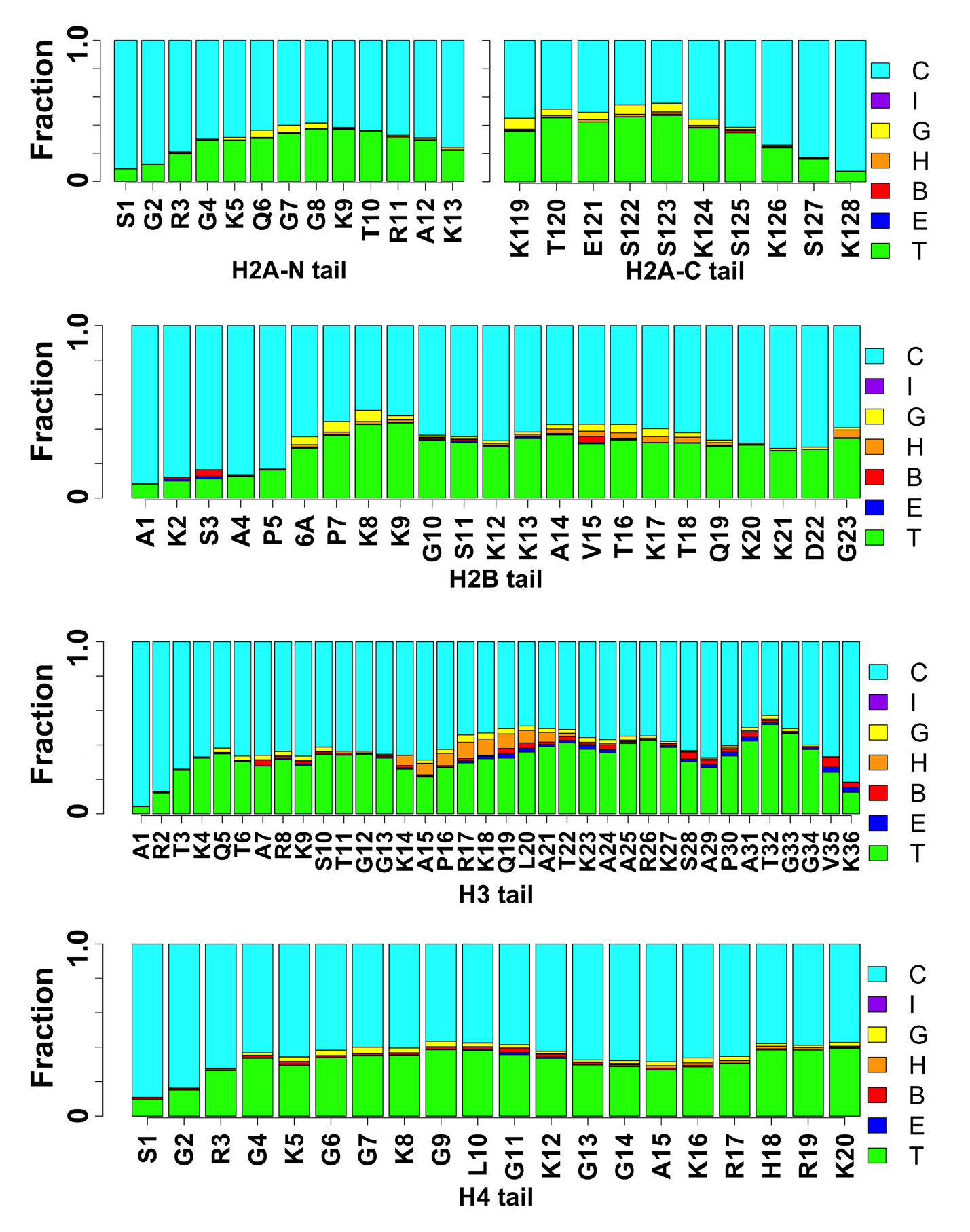
**

**Supplementary Figure 6.** Characterization of histone H2A C-terminal tail bound and unbound states in different simulation runs. Models A, B, C, and D have different initial tail configurations (described in Supplementary Table 1). The tail conformation per frame is categorized into unbound, intermediately bound, and strongly bound states. Red and blue lines indicate the conformational changes of two copies of histone tails in nucleosome. Unbound state is defined if the percentage of tail residues maintaining contacts with DNA is no more than the cut-offs, while the strongly bound state is defined if the percentage of residues bound with DNA is more than or equal to (one minus cut-off). Tails in the intermediate bound state have the percentage of residues interacting with DNA between cut-off and (one minus cut-off).


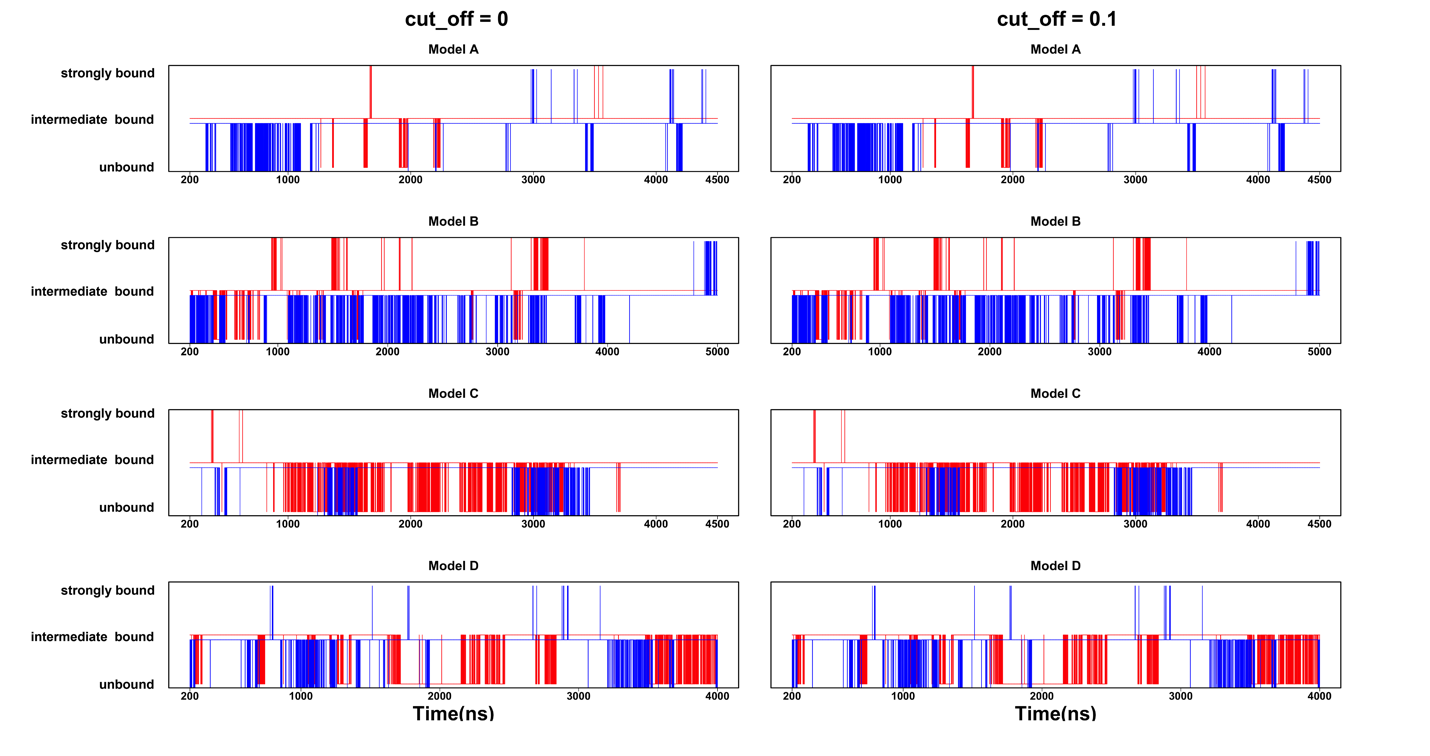
**Supplementary Figure 7.** Characterization of histone H2A N-terminal tail bound and unbound states in different simulation runs. Models A, B, C, and D have different initial tail configurations (described in Supplementary Table 1). The tail conformation per frame is categorized into unbound, intermediately bound, and strongly bound states. Red and blue lines indicate the conformational changes of two copies of histone tails in nucleosome. Unbound state is defined if the percentage of tail residues maintaining contacts with DNA is no more than the cut-offs, while the strongly bound state is defined if the percentage of residues bound with DNA is more than or equal to (one minus cut-off). Tails in the intermediate bound state have the percentage of residues interacting with DNA between cut-off and (one minus cut-off).


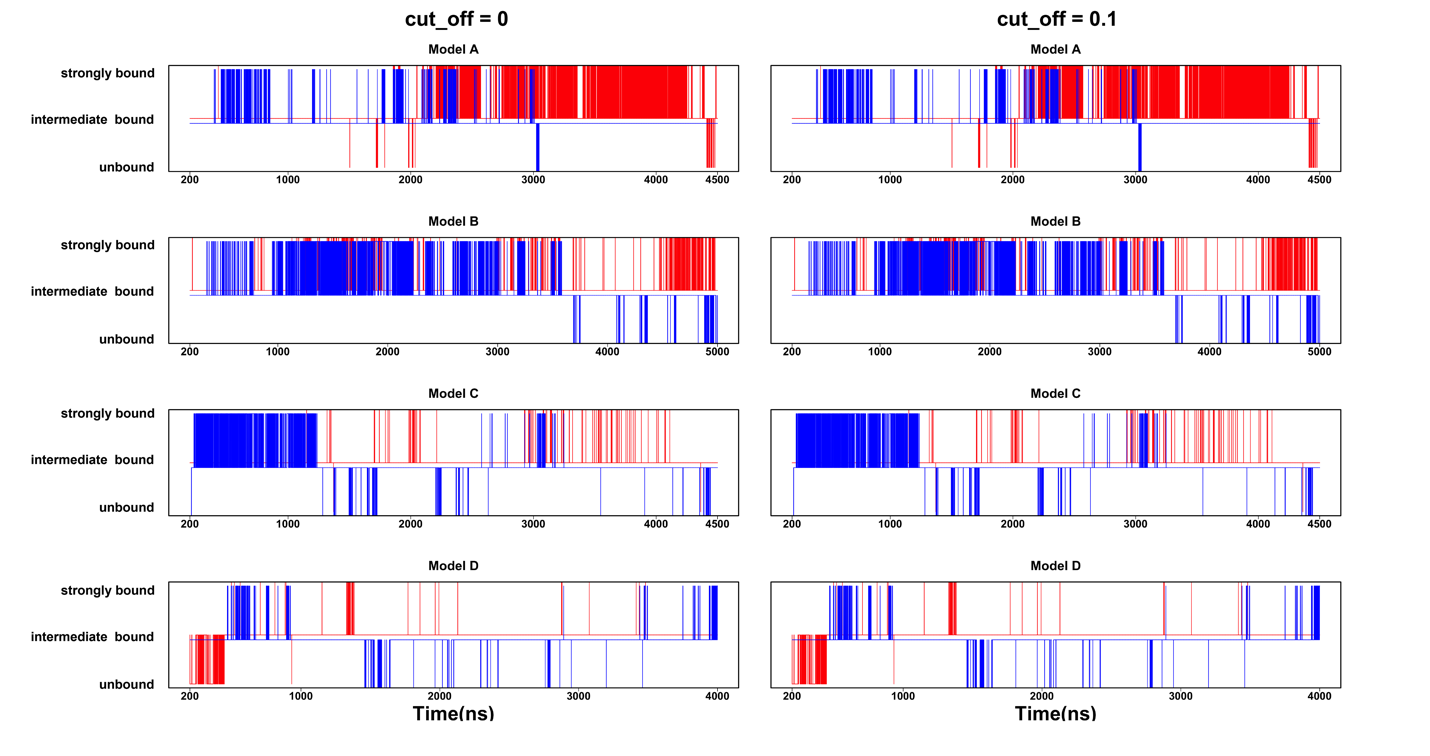
**Supplementary Figure 8.** Characterization of histone H2B tail bound and unbound states in different simulation runs. Models A, B, C, and D have different initial tail configurations (described in Supplementary Table 1). The tail conformation per frame is categorized into unbound, intermediately bound, and strongly bound states. Red and blue lines indicate the conformational changes of two copies of histone tails in nucleosome. Unbound state is defined if the percentage of tail residues maintaining contacts with DNA is no more than the cut-offs, while the strongly bound state is defined if the percentage of residues bound with DNA is more than or equal to (one minus cut-off). Tails in the intermediate bound state have the percentage of residues interacting with DNA between cut-off and (one minus cut-off).


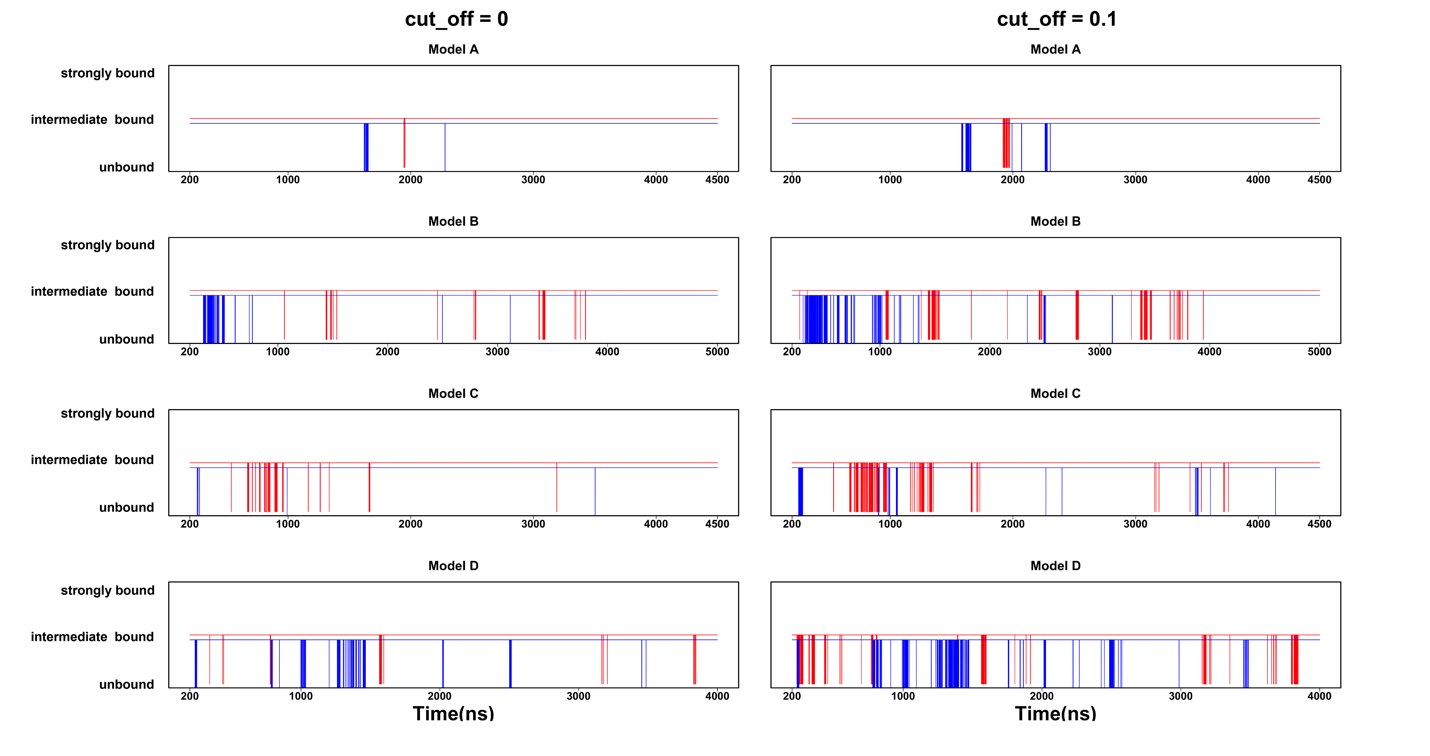


**Supplementary Figure 9.** Characterization of histone H3 tail bound and unbound states in different simulation runs. Models A, B, C, and D have different initial tail configurations (described in Supplementary Table 1). The tail conformation per frame is categorized into unbound, intermediately bound, and strongly bound states. Red and blue lines indicate the conformational changes of two copies of histone tails in nucleosome. Unbound state is defined if the percentage of tail residues maintaining contacts with DNA is no more than the cut-offs, while the strongly bound state is defined if the percentage of residues bound with DNA is more than or equal to (one minus cut-off). Tails in the intermediate bound state have the percentage of residues interacting with DNA between cut-off and (one minus cut-off).


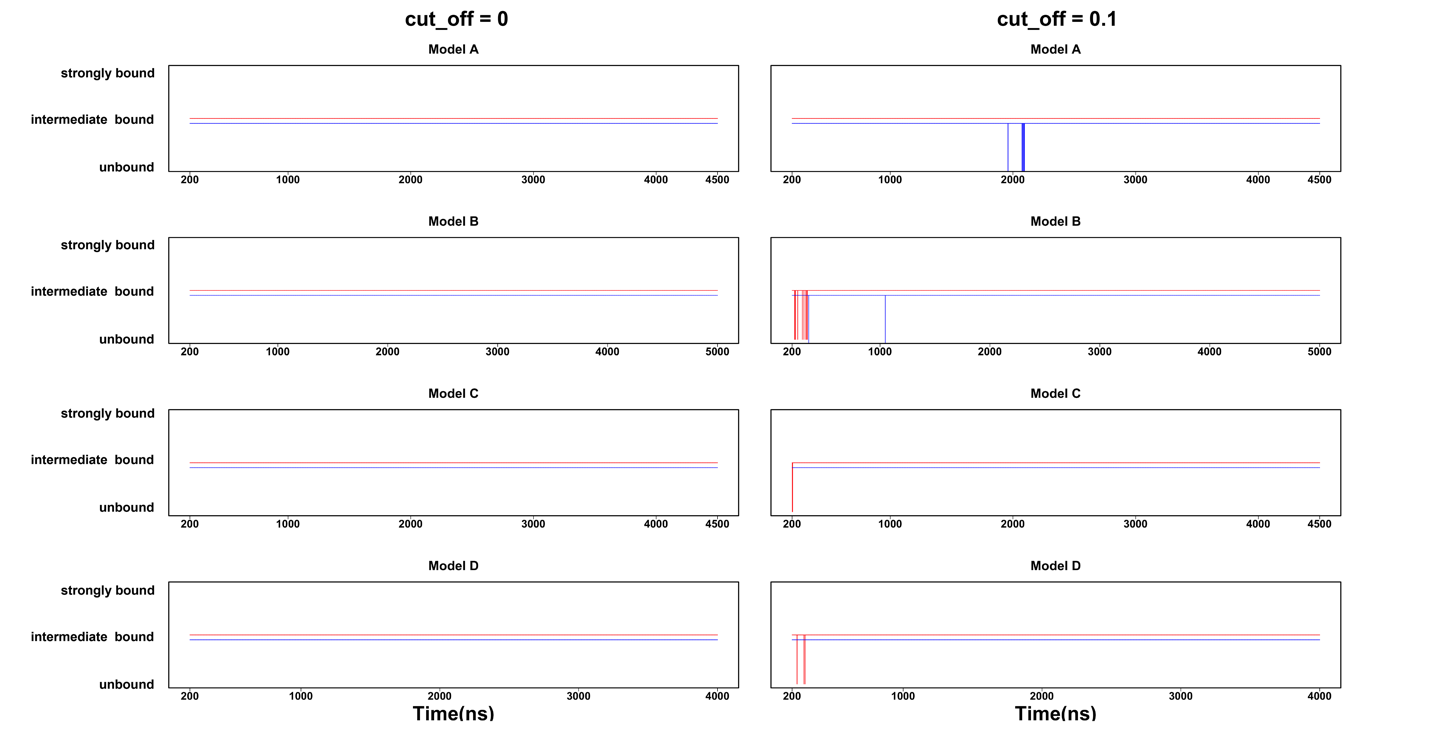
**Supplementary Figure 10.** Characterization of histone H4 tail bound and unbound states in different simulation runs. Models A to D have different initial tail configurations (described in Supplementary Table 1). The tail conformation per frame is categorized into unbound, intermediately bound, and strongly bound states. Red and blue lines indicate the conformational changes of two copies of histone tails in nucleosome. Unbound state is defined if the percentage of tail residues maintaining contacts with DNA is no more than the cut-offs, while the strongly bound state is defined if the percentage of residues bound with DNA is more than or equal to (one minus cut-off). Tails in the intermediate bound state have the percentage of residues interacting with DNA between cut-off and (one minus cut-off).


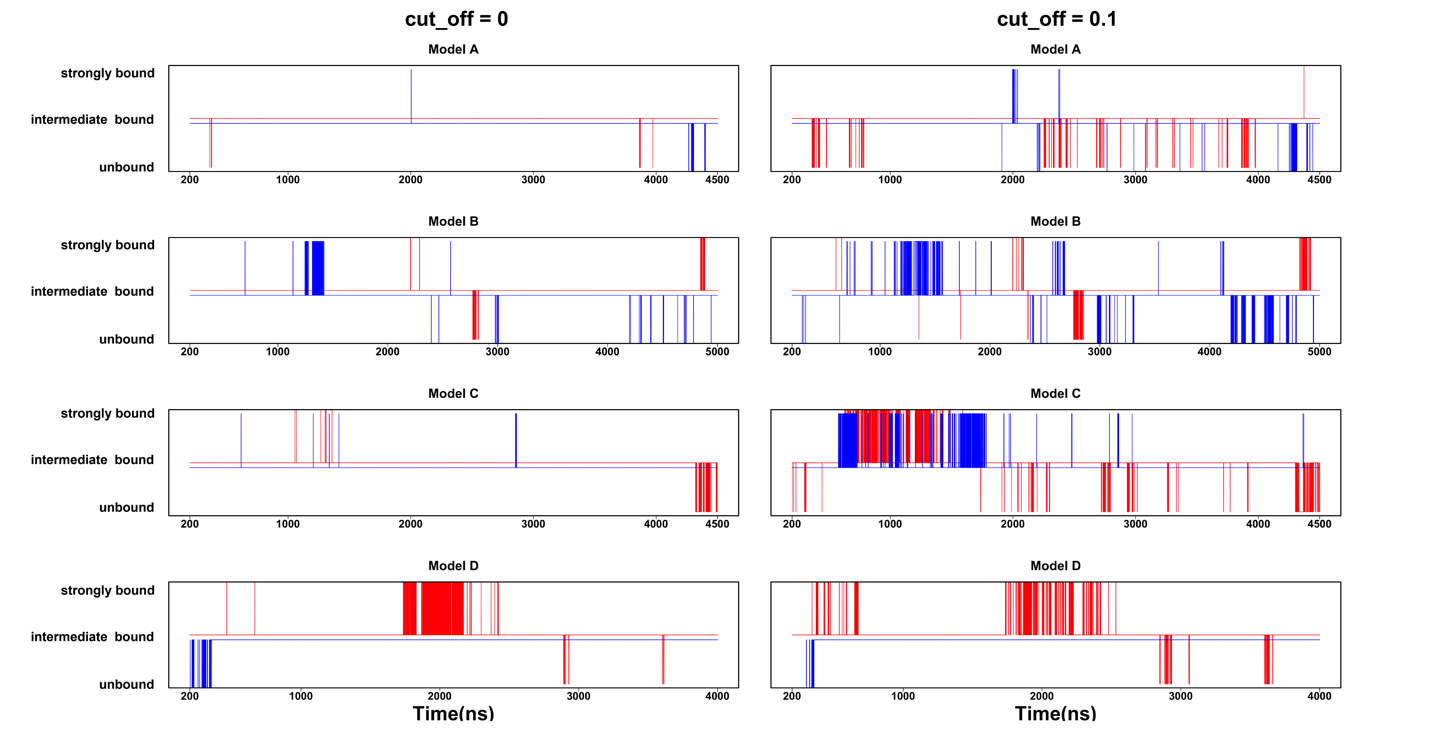
**Supplementary Figure 11.** Mean number of contacts between histone tails and DNA per histone type. The numbers are averaged and error bars represent the standard error of the mean from independent simulation runs (n=22). Source data are provided as a Source Data file.


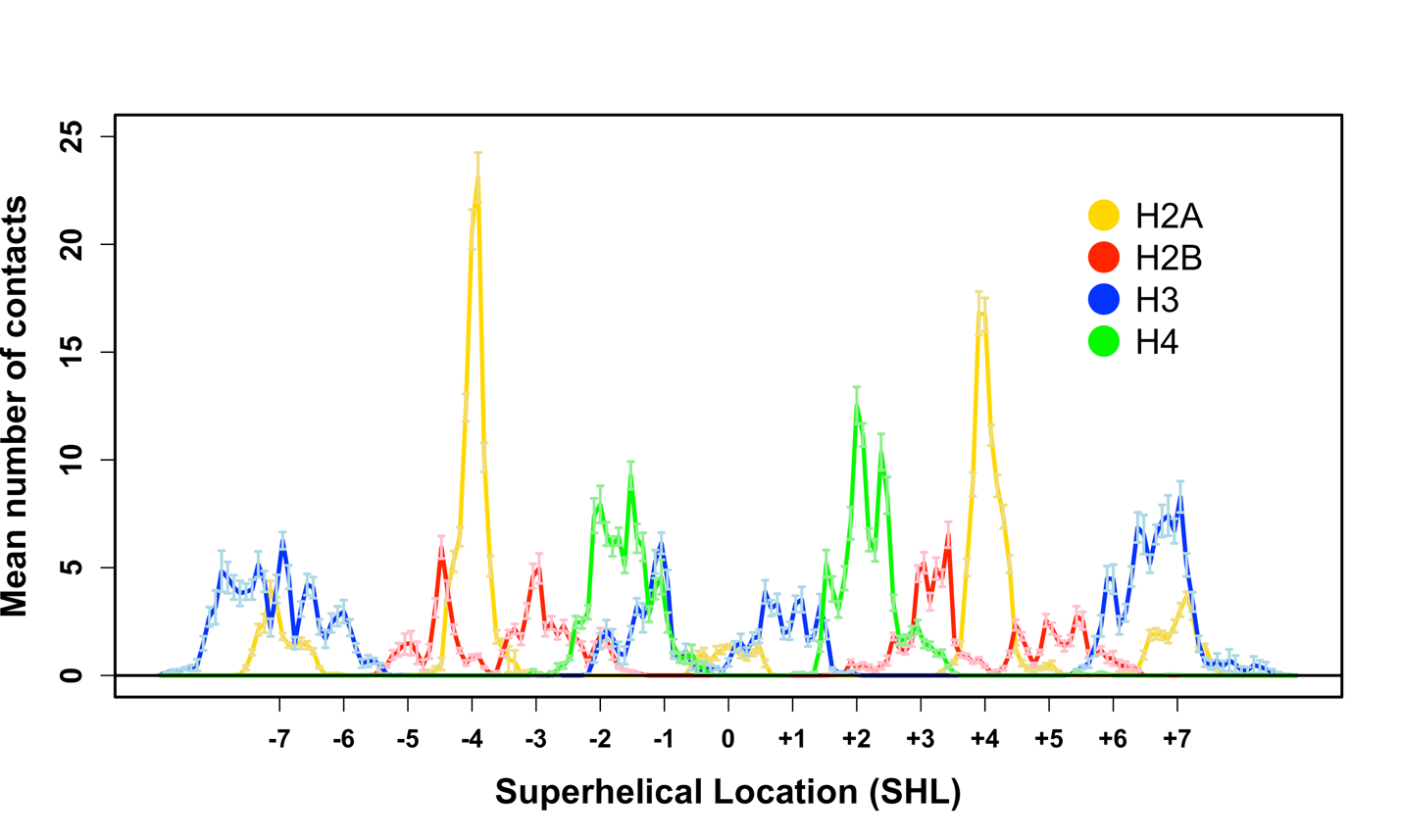


**Supplementary Figure 12.** The Pearson correlation coefficient calculated between the mean numbers of tail-DNA contacts for DNA regions occupied by both copies of histone tails (SHL+ and SHL-). Two-tailed t-test was performed. Source data are provided as a Source Data file.

**
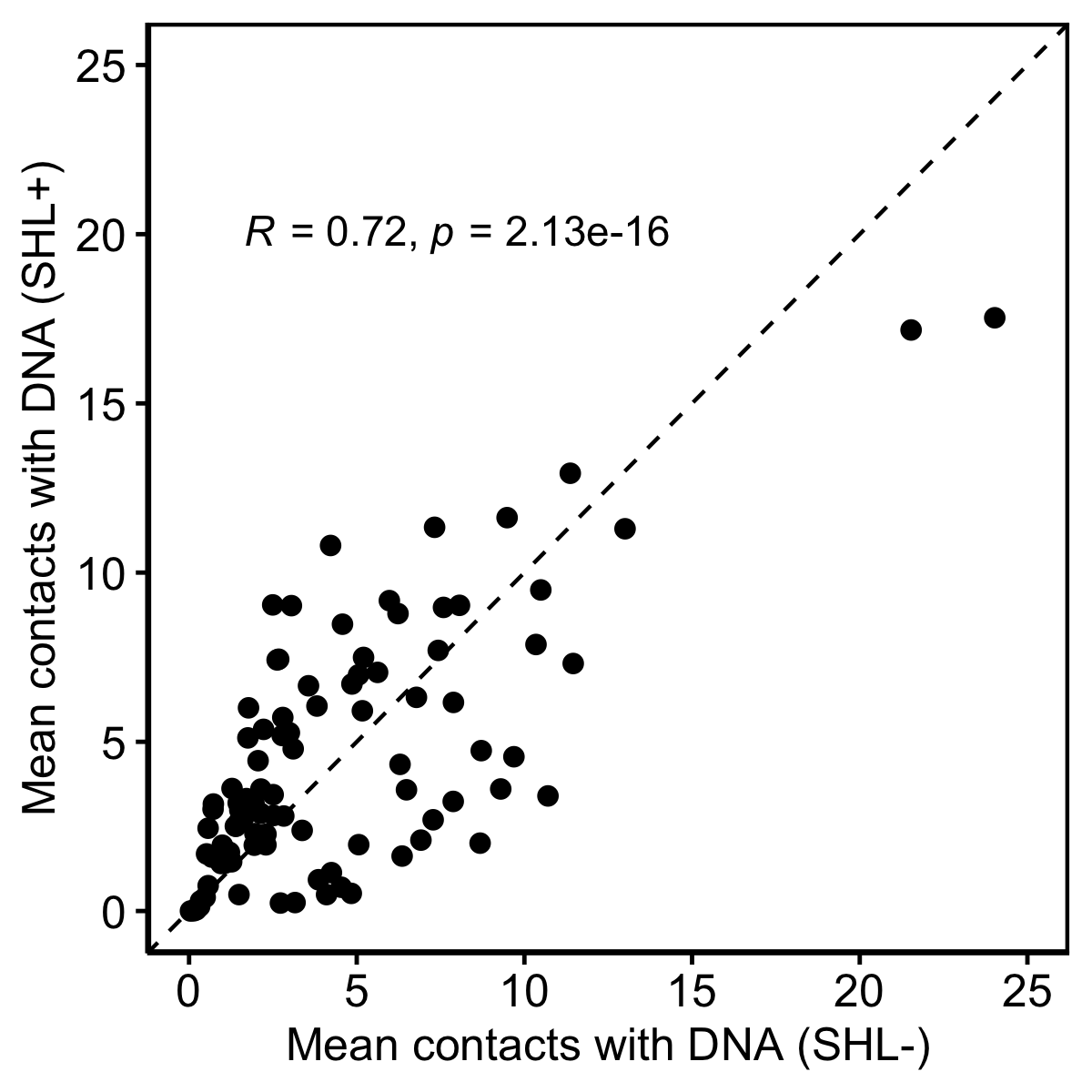
**

**Supplementary Figure 13.** Mean numbers of tail-DNA contacts combined from all types of histone tails per model. The numbers are averaged over 1ns frames of simulation runs per model. Source data are provided as a Source Data file.

**
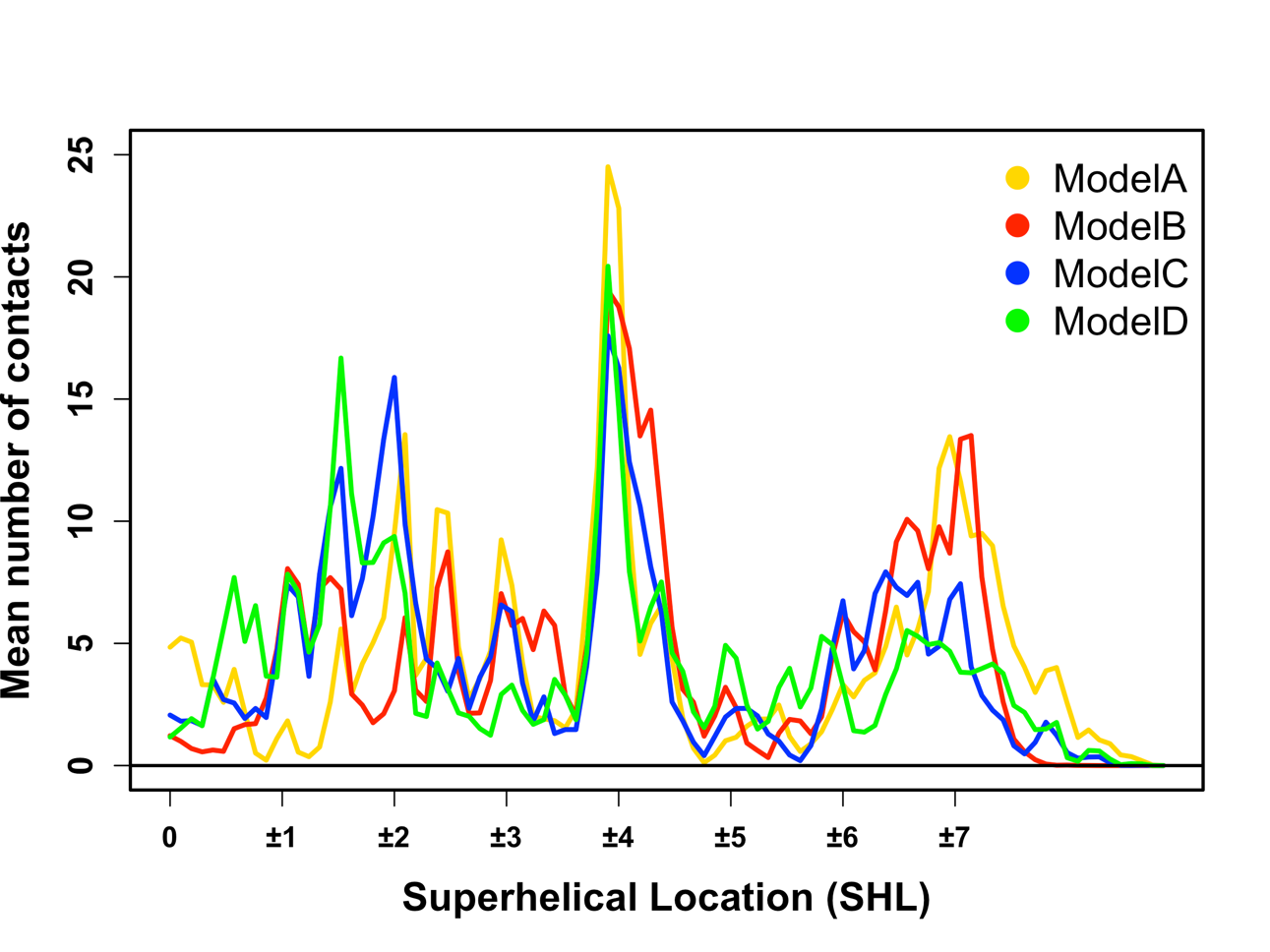
**

**Supplementary Figure 14.** The change of DNA accessibility is highly correlated with the number of contacts between DNA and tails. a) Changes of DNA solvent accessibility surface area (SASA) upon histone tail binding (units in Å^2^). The numbers are averaged, and error bars represent the standard error of the mean from independent simulation runs for two copies (n=44). b) Mean number of tail-DNA contacts over all types of histone tails from independent simulation runs for two copies (n=44). The number of contacts of two copies of histone tails are combined due to the 2-fold pseudo-symmetry of the nucleosome structure. The error bars represent standard errors of the mean. c) The Pearson correlation coefficient calculated between the mean number of tail-DNA contacts and the mean value of the DNA SASA change per DNA base pair. Two-tailed t-test is performed. Source data are provided as a Source Data file.


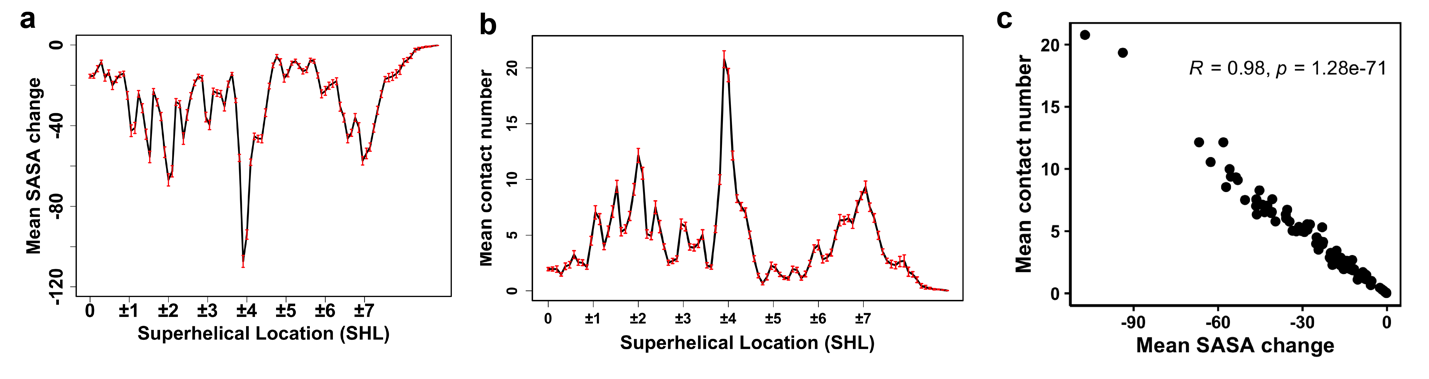


**Supplementary Figure 15.** Thermodynamic cycle to estimate the binding free energy of protein to tail-DNA complex. ΔG(Pro-Tail-DNA), ΔG(Tail-DNA), ΔG(Pro-DNA), and ΔG(Tail-Pro) represent binding free energies of protein to tail-DNA complex, histone tail to free DNA, protein to free DNA, and histone tail to protein-DNA complex.


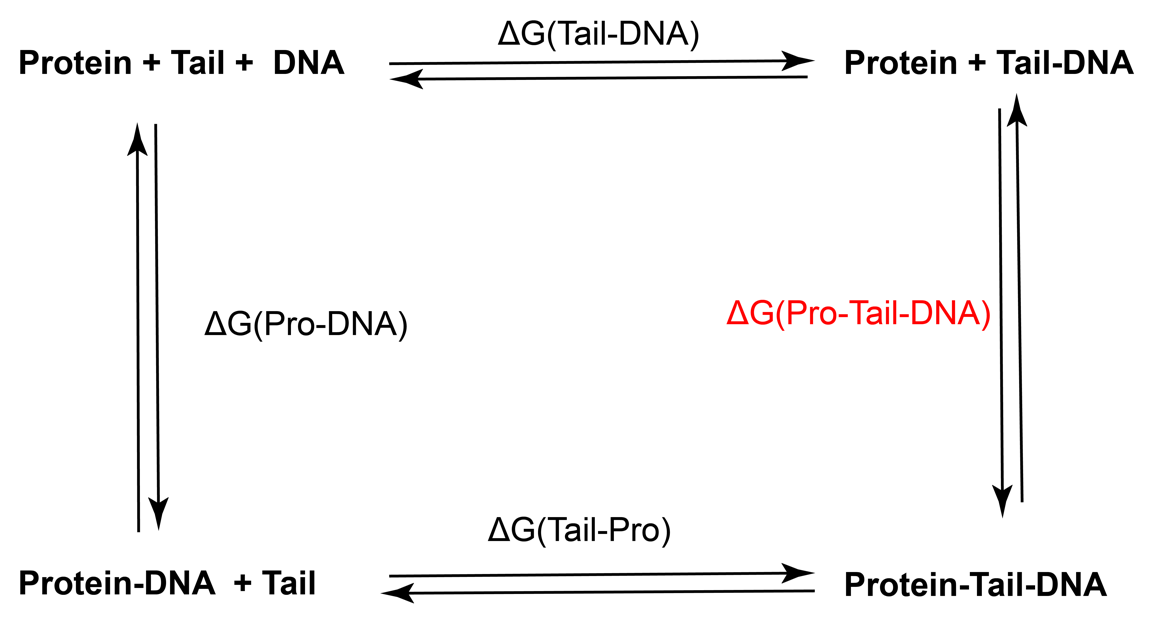


**Supplementary Figure 16.** Numbers of cancer-associated mutations (pink) and binding proteins (light blue) mapped on tracks representing consensus sequence of the full alignment of histone sequences (see Supplementary Fig. 17, 18, 19, 20). Globular domains are indicated as yellow, red, blue and green bars per histone type. Black asterisks denote the acidic patch residues.

**
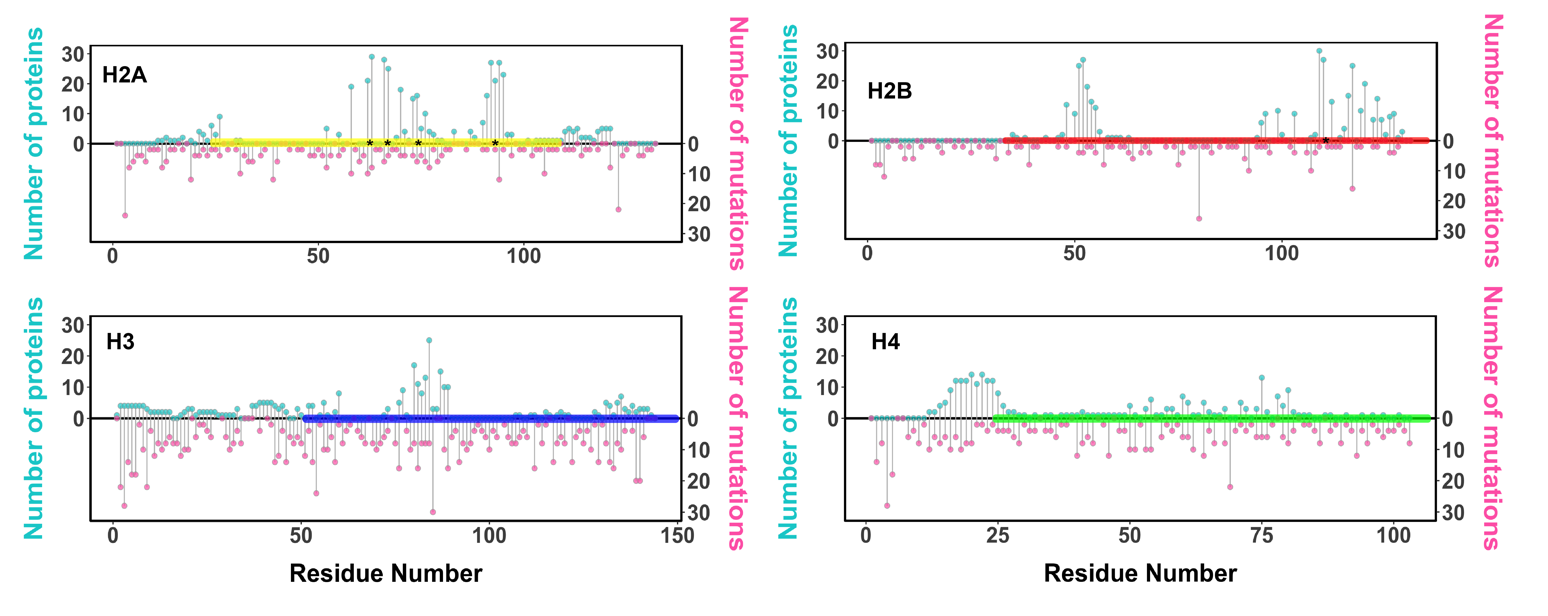
**

**Supplementary Figure 17**. Multiple sequence alignment of all H2A proteins present in nucleosome complex structures. Histone sequences are extracted from HistoneDB 2.0 ^21^ and aligned using Clustal Omega 1.2.3 ^22^. The highly conserved residues are colored red.

**Supplementary Figure 18.** Multiple sequence alignment of all H2B proteins present in nucleosome complex structures. Histone sequences are extracted from HistoneDB 2.0 ^21^ and aligned using Clustal Omega 1.2.3 ^22^. The highly conserved residues are colored red.

**Supplementary Figure 19.** Multiple sequence alignment of all H3 proteins present in nucleosome complex structures. Histone sequences are extracted from HistoneDB 2.0 ^21^ and aligned using Clustal Omega 1.2.3 ^22^. The highly conserved residues are colored red.

**Supplementary Figure 20.** Multiple sequence alignment of all H4 proteins present in nucleosome complex structures. Histone sequences are extracted from HistoneDB 2.0 ^21^ and aligned using Clustal Omega 1.2.3 ^22^. The highly conserved residues are colored red.

**Supplementary Figure 21.** Comparison of histone tail full residence time ($\tau_{f}$) between unmodified and modified tails. Simulations of model D with and without tail modifications are used for analysis. The unbinding of tails is defined if the percentage of tail residues maintaining contacts with DNA is no more than 10%. $\tau_{f}$ of unmodified tails were calculated using the first 1600ns trajectory from the simulation of Model D with AMBER package (Supplementary Table 1). Box-plot elements are defined as: center line, median; box limits, upper and lower quartiles; whiskers are drawn at values equal to 1.5× interquartile range; Unmodified Tail: n(H2A_N) = 6, n(H2A_C) = 30, n(H2B) = 24, n(H3) = 4, n(H4) = 2; Arg->Ala Mutation: n(H2A_N) = 32, n(H2A_C) = 30, n(H2B) = 17, n(H3) = 17, n(H4) = 38; Lys Acetylation: n(H2A_N) = 12, n(H2A_C) = 15, n(H2B) = 11, n(H3) = 17, n(H4) = 10; Lys Trimethylation: n(H2A_N) = 10, n(H2A_C) = 25, n(H2B) = 16, n(H3) = 5, n(H4) = 12; Ser/Thr Phosphorylation: n(H2A_N) = 8, n(H2A_C) = 8, n(H2B) = 19, n(H3) = 11, n(H4) = 21; Single Modification per Tail: n(H2A_N) = 13, n(H2A_C) = 23, n(H2B) = 20, n(H3) = 13, n(H4) = 5; Source data are provided as a Source Data file.


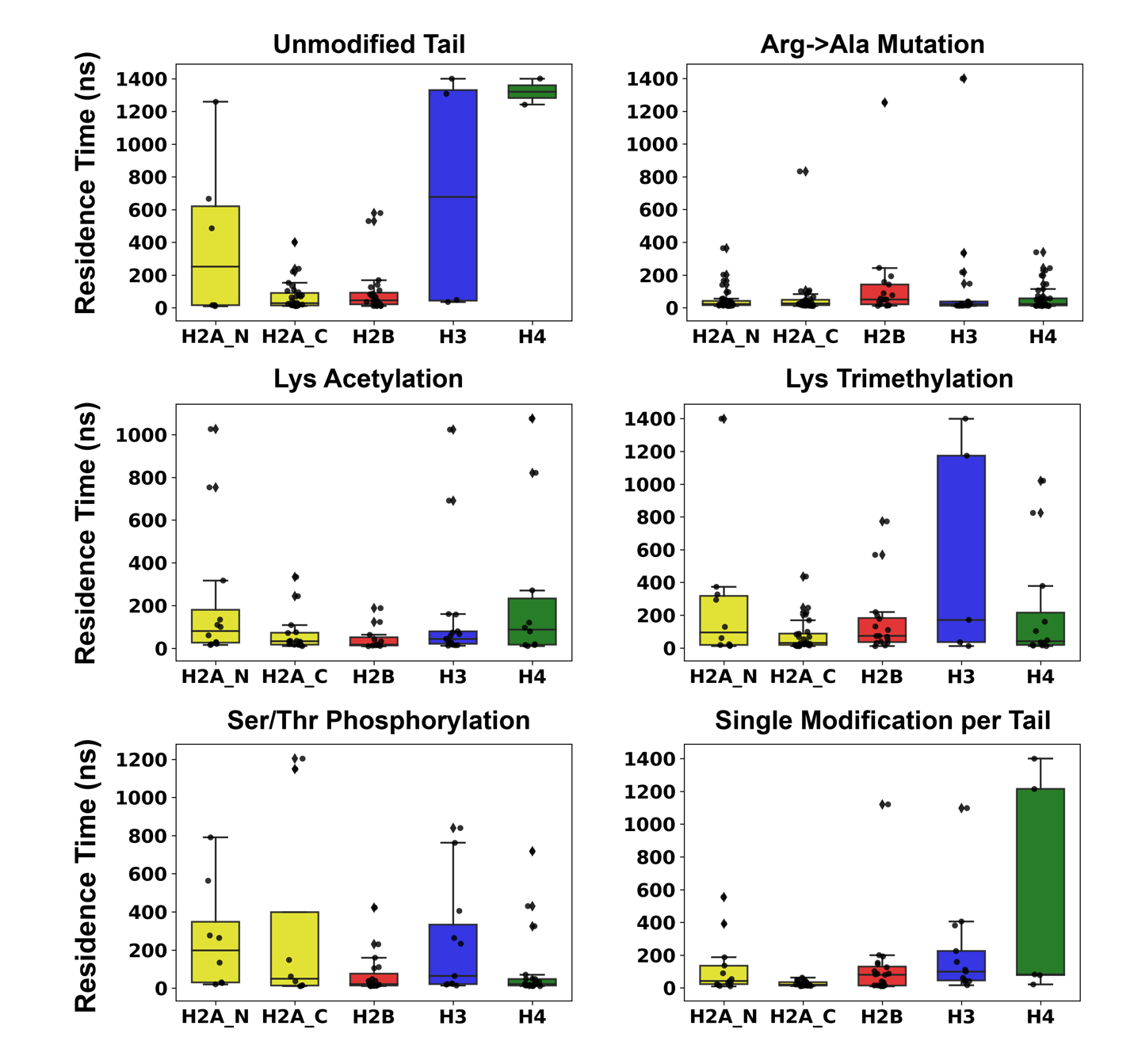


**Supplementary Figure 22.** The change of the mean number of contacts between histone tails and nucleosomal and linker DNA per base pair upon modifications. For each modification type, the change of the mean number of contacts per DNA base pair is calculated as the mean number of contacts between DNA and modified tails minus the mean number of contacts between DNA and unmodified tails. The reported values are averaged, and the error bars represent the standard errors of the mean for modified tails calculated from independent simulation runs (n=10). One-tailed t-test has been performed for analyzing the statistical significance of changes of the full tail-DNA contacts upon modifications. The null hypothesis is that the mean change of tail-DNA contacts upon modifications is zero. The alternative hypothesis is that tail modification decreases the overall mean contact number between tail and DNA. Table lists p-values, if p-value is less than 0.05 the change is considered to be significant for the full tail. For each DNA site significant changes occur if the error bars do not extend beyond zero. Source data are provided as a Source Data file.

**
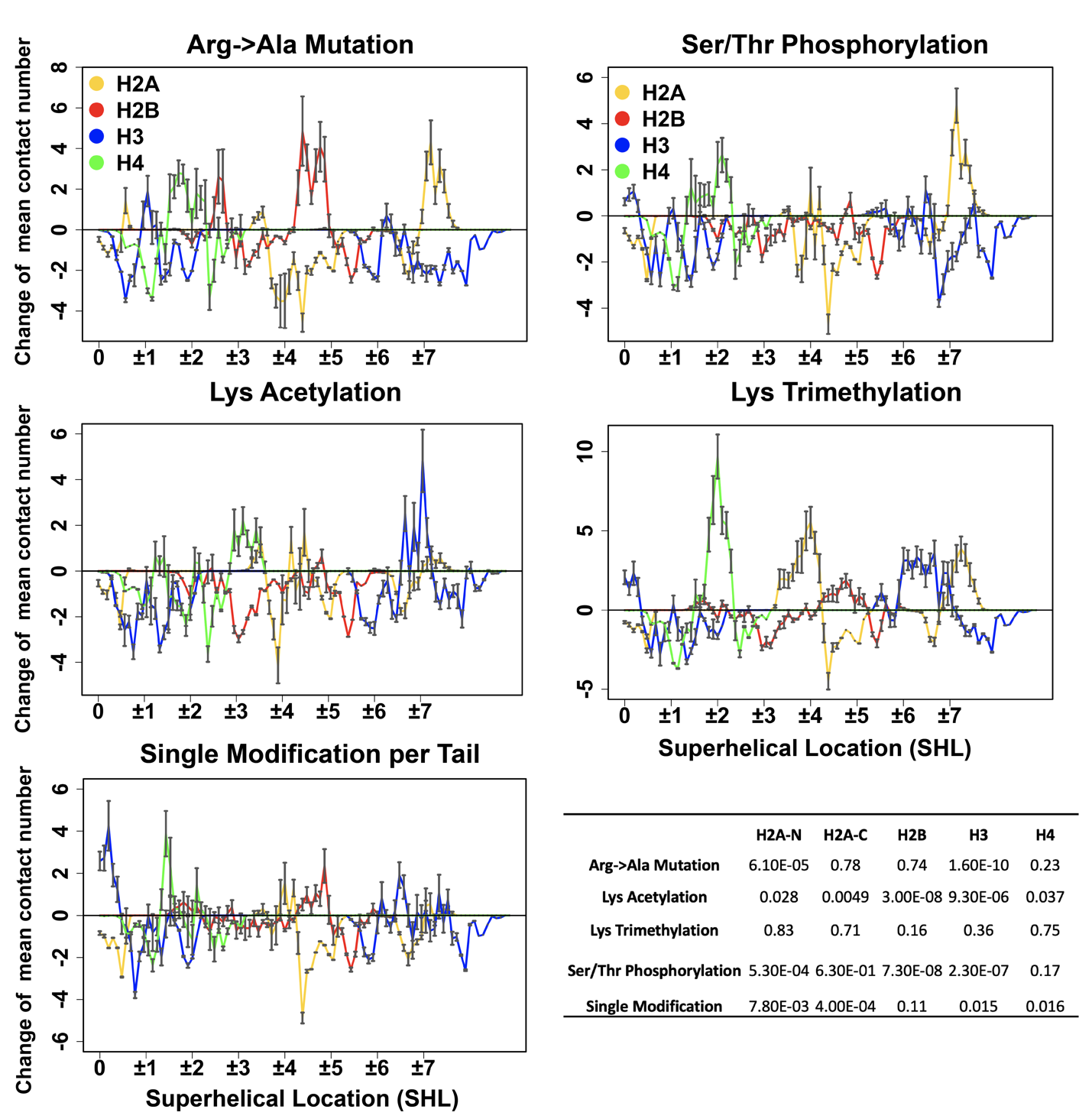
**

**Supplementary Figure 23.** Standard state protein-DNA binding free energies taken from dbAMEPNI database^16^. The distribution of experimentally determined binding free energies for 83 DNA binding proteins. The density distribution is smoothed using the gaussian smoothing kernel. Source data are provided as a Source Data file.


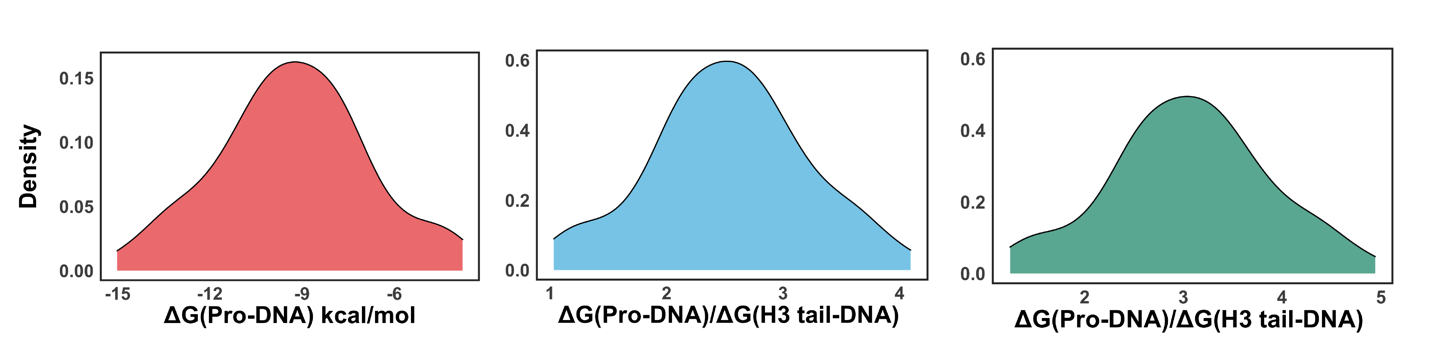


**Supplementary Reference**

1 Piana, S., Donchev, A. G., Robustelli, P. & Shaw, D. E. Water dispersion interactions strongly influence simulated structural properties of disordered protein states. *J Phys Chem B* **119**, 5113-5123, doi:10.1021/jp508971m (2015).

2 Shabane, P. S. & Onufriev, A. V. Significant compaction of H4 histone tail upon charge neutralization by acetylation and its mimics, possible effects on chromatin structure. *J Mol Biol*, 166683, doi:10.1016/j.jmb.2020.10.017 (2020).

3 Shabane, P. S., Izadi, S. & Onufriev, A. V. General Purpose Water Model Can Improve Atomistic Simulations of Intrinsically Disordered Proteins. *J Chem Theory Comput* **15**, 2620-2634, doi:10.1021/acs.jctc.8b01123 (2019).

4 Bergonzo, C. & Cheatham, T. E., 3rd. Improved Force Field Parameters Lead to a Better Description of RNA Structure. *J Chem Theory Comput* **11**, 3969-3972, doi:10.1021/acs.jctc.5b00444 (2015).

5 Dans, P. D. *et al.* Modeling, Simulations, and Bioinformatics at the Service of RNA Structure. *Chem* **5**, 51-73, doi:10.1016/j.chempr.2018.09.015 (2019).

6 Kuhrova, P. *et al.* Improving the Performance of the Amber RNA Force Field by Tuning the Hydrogen-Bonding Interactions. *J Chem Theory Comput* **15**, 3288-3305, doi:10.1021/acs.jctc.8b00955 (2019).

7 Galindo-Murillo, R. *et al.* Assessing the Current State of Amber Force Field Modifications for DNA. *J Chem Theory Comput* **12**, 4114-4127, doi:10.1021/acs.jctc.6b00186 (2016).

8 Yang, C., Kulkarni, M., Lim, M. & Pak, Y. Insilico direct folding of thrombin-binding aptamer G-quadruplex at all-atom level. *Nucleic Acids Res* **45**, 12648-12656, doi:10.1093/nar/gkx1079 (2017).

9 Zhou, B. R. *et al.* Distinct Structures and Dynamics of Chromatosomes with Different Human Linker Histone Isoforms. *Mol Cell* **81**, 166-182 e166, doi:10.1016/j.molcel.2020.10.038 (2021).

10 Gatchalian, J. *et al.* Accessibility of the histone H3 tail in the nucleosome for binding of paired readers. *Nat Commun* **8**, 1489, doi:10.1038/s41467-017-01598-x (2017).

11 Morrison, E. A., Bowerman, S., Sylvers, K. L., Wereszczynski, J. & Musselman, C. A. The conformation of the histone H3 tail inhibits association of the BPTF PHD finger with the nucleosome. *Elife* **7**, doi:10.7554/eLife.31481 (2018).

12 Shaytan, A. K. *et al.* Coupling between Histone Conformations and DNA Geometry in Nucleosomes on a Microsecond Timescale: Atomistic Insights into Nucleosome Functions. *J Mol Biol* **428**, 221-237, doi:10.1016/j.jmb.2015.12.004 (2016).

13 Humphrey, W., Dalke, A. & Schulten, K. VMD: visual molecular dynamics. *J Mol Graph* **14**, 33-38, 27-38, doi:10.1016/0263-7855(96)00018-5 (1996).

14 Phillips, J. C. *et al.* Scalable molecular dynamics with NAMD. *J Comput Chem* **26**, 1781-1802, doi:10.1002/jcc.20289 (2005).

15 Pilotto, S. *et al.* Interplay among nucleosomal DNA, histone tails, and corepressor CoREST underlies LSD1-mediated H3 demethylation. *Proc Natl Acad Sci U S A* **112**, 2752-2757, doi:10.1073/pnas.1419468112 (2015).

16 Liu, L. *et al.* dbAMEPNI: a database of alanine mutagenic effects for protein-nucleic acid interactions. *Database (Oxford)* **2018**, doi:10.1093/database/bay034 (2018).

17 Bonnet, J. *et al.* Quantification of Proteins and Histone Marks in Drosophila Embryos Reveals Stoichiometric Relationships Impacting Chromatin Regulation. *Dev Cell* **51**, 632-644 e636, doi:10.1016/j.devcel.2019.09.011 (2019).

18 Milo, R. & Phillips, R. *Cell biology by the numbers*. (Garland Science, 2015).

19 Langst, G. & Manelyte, L. Chromatin Remodelers: From Function to Dysfunction. *Genes (Basel)* **6**, 299-324, doi:10.3390/genes6020299 (2015).

20 Makowski, M. M. *et al.* Global profiling of protein-DNA and protein-nucleosome binding affinities using quantitative mass spectrometry. *Nat Commun* **9**, 1653, doi:10.1038/s41467-018-04084-0 (2018).

21 Draizen, E. J. *et al.* HistoneDB 2.0: a histone database with variants--an integrated resource to explore histones and their variants. *Database (Oxford)* **2016**, doi:10.1093/database/baw014 (2016).

22 Sievers, F. & Higgins, D. G. Clustal Omega for making accurate alignments of many protein sequences. *Protein Sci* **27**, 135-145, doi:10.1002/pro.3290 (2018).
