# Supplementary material for: Binding of regulatory proteins to nucleosomes is modulated by dynamic histone tails
Source: Nat Commun. 2021 Sep 6;12:5280. doi: 10.1038/s41467-021-25568-6 (PMC8421395; doi:10.1038/s41467-021-25568-6)
Supplement: Supplementary file 3 — Reporting Summary [file 41467_2021_25568_MOESM3_ESM.pdf]

nature portfolio

Corresponding author(s): Anna Panchenko

Last updated by author(s): Jul 15, 2021

Reporting Summary

Nature Portfolio wishes to improve the reproducibility of the work that we publish. This form provides structure for consistency and transparency in reporting. For further information on Nature Portfolio policies, see our Editorial Policies and the Editorial Policy Checklist.

Statistics

For all statistical analyses, confirm that the following items are present in the figure legend, table legend, main text, or Methods section.

n/a	Confirmed	
☐	☒	The exact sample size (*n*) for each experimental group/condition, given as a discrete number and unit of measurement
☐	☒	A statement on whether measurements were taken from distinct samples or whether the same sample was measured repeatedly
☐	☒	The statistical test(s) used AND whether they are one- or two-sided<br>*Only common tests should be described solely by name; describe more complex techniques in the Methods section.*
☒	☐	A description of all covariates tested
☒	☐	A description of any assumptions or corrections, such as tests of normality and adjustment for multiple comparisons
☐	☒	A full description of the statistical parameters including central tendency (e.g. means) or other basic estimates (e.g. regression coefficient) AND variation (e.g. standard deviation) or associated estimates of uncertainty (e.g. confidence intervals)
☐	☒	For null hypothesis testing, the test statistic (e.g. *F*, *t*, *r*) with confidence intervals, effect sizes, degrees of freedom and *P* value noted<br>*Give P values as exact values whenever suitable.*
☒	☐	For Bayesian analysis, information on the choice of priors and Markov chain Monte Carlo settings
☒	☐	For hierarchical and complex designs, identification of the appropriate level for tests and full reporting of outcomes
☒	☐	Estimates of effect sizes (e.g. Cohen's *d*, Pearson's *r*), indicating how they were calculated

Our web collection on statistics for biologists contains articles on many of the points above.

Software and code

Policy information about availability of computer code

Data collection	Multiple molecular dynamic simulation runs were performed using the Amber18 (https://ambermd.org), GROMACS version 2019.3 (http://www.gromacs.org) and NAMD2.12 (https://www.ks.uiuc.edu/Research/namd/).
Data analysis	Custom Python3 scripts using the following key libraries: numpy1.18.1, pandas1.0.1, requests2.22.0<br>VMD version 1.9.3 available at https://www.ks.uiuc.edu/Research/vmd/<br>Custom R scripts for making plots<br>Bwtool version 1.0 available at https://github.com/CRG-Barcelona/bwtool<br>Chimera version 1.13.1 available at https://www.cgl.ucsf.edu/chimera/download.html<br>3DNA version 2.4 available at https://x3dna.org<br>Dephi version 8.4.5 available at http://compbio.clemson.edu/lab/delphisw/<br>All the scripts, source data and instructions are available at https://github.com/Panchenko-Lab/Supplementary-data-for-Peng-et-al-2021

For manuscripts utilizing custom algorithms or software that are central to the research but not yet described in published literature, software must be made available to editors and reviewers. We strongly encourage code deposition in a community repository (e.g. GitHub). See the Nature Portfolio guidelines for submitting code & software for further information.

Data

Policy information about availability of data

All manuscripts must include a data availability statement. This statement should provide the following information, where applicable:

- Accession codes, unique identifiers, or web links for publicly available datasets
- A description of any restrictions on data availability
- For clinical datasets or third party data, please ensure that the statement adheres to our policy

Source data are provided with this paper and available from GitHub at https://github.com/Panchenko-Lab/Supplementary-data-for-Peng-et-al-2021. Molecular dynamics simulation trajectories generated in this study are archived via Zenodo at https://doi.org/10.5281/zenodo.4771269. Fragments of 147 bp lengths of high-coverage MNase-seq reads used in this study are available in the GEO under the accession number GSE36979. Nucleosome structures used in this study are available in Protein Data Bank (https://www.rcsb.org).

Field-specific reporting

Please select the one below that is the best fit for your research. If you are not sure, read the appropriate sections before making your selection.

[x] Life sciences [ ] Behavioural & social sciences [ ] Ecological, evolutionary & environmental sciences

For a reference copy of the document with all sections, see nature.com/documents/nr-reporting-summary-flat.pdf

Life sciences study design

All studies must disclose on these points even when the disclosure is negative.

Sample size	We did not apply one statistical method to predetermine the sample size since this is not applicable to simulation studies. The sample size (simulation time and number of simulation runs) are determined by the convergence of the simulation runs and variances of measurements. Our results show that sampling of tail conformations is convergent and the measured values vary little among different independent runs. This can indicate the sufficient sample size of our studies.
Data exclusions	No data was excluded.
Replication	For each nucleosome model, we performed five replicated simulation runs. In the analysis, we calculated the standard error of mean from each independent runs and show that our results do not vary significantly among replicated runs.
Randomization	Randomization is not relevant to our study since we do not allocate samples into different groups. The data from simulations are not applicable for randomization and can be directly used for analysis.
Blinding	Blinding test is not relevant to our study since we do not collect statistical sample. All the data from simulations are not applicable for group allocation and can be directly used for analysis.

Reporting for specific materials, systems and methods

We require information from authors about some types of materials, experimental systems and methods used in many studies. Here, indicate whether each material, system or method listed is relevant to your study. If you are not sure if a list item applies to your research, read the appropriate section before selecting a response.

Materials & experimental systems

n/a	Involved in the study	
[x]	[ ]	Antibodies
[x]	[ ]	Eukaryotic cell lines
[x]	[ ]	Palaeontology and archaeology
[x]	[ ]	Animals and other organisms
[x]	[ ]	Human research participants
[x]	[ ]	Clinical data
[x]	[ ]	Dual use research of concern

Methods

n/a	Involved in the study	
[x]	[ ]	ChIP-seq
[x]	[ ]	Flow cytometry
[x]	[ ]	MRI-based neuroimaging
